# Asymmetry in responsiveness to playback of geographic song variation after a recent range expansion in light-vented bulbuls

**DOI:** 10.1093/beheco/arag012

**Published:** 2026-02-10

**Authors:** Xiaoying Xing, Xiaojing Yang, Xiajuan Xu, Changjian Fu, Gang Song, Fumin Lei, Hans Slabbekoorn

**Affiliations:** College of Wildlife and Protected Area, Northeast Forestry University, Hexing Road No. 26, Xiangfang District, Harbin, Heilongjiang 150040, China; Northeast Asia Biodiversity Research Center, No. 26 Hexing Road, Xiangfang District, Harbin, Heilongjiang 150040, China; School of Environmental Studies, China University of Geosciences, No. 388 Lumo Road, Hongshan District, Wuhan, Hubei 430074, China; College of Wildlife and Protected Area, Northeast Forestry University, Hexing Road No. 26, Xiangfang District, Harbin, Heilongjiang 150040, China; Northeast Asia Biodiversity Research Center, No. 26 Hexing Road, Xiangfang District, Harbin, Heilongjiang 150040, China; College of Wildlife and Protected Area, Northeast Forestry University, Hexing Road No. 26, Xiangfang District, Harbin, Heilongjiang 150040, China; Northeast Asia Biodiversity Research Center, No. 26 Hexing Road, Xiangfang District, Harbin, Heilongjiang 150040, China; Key Laboratory of Zoological Systematics and Evolution, Institute of Zoology, Chinese Academy of Sciences, No. 1 Beichen West Road, Chaoyang District, Beijing 100101, China; Key Laboratory of Zoological Systematics and Evolution, Institute of Zoology, Chinese Academy of Sciences, No. 1 Beichen West Road, Chaoyang District, Beijing 100101, China; College of Life Science, University of Chinese Academy of Sciences, No. 19A Yuquan Road, Shijingshan District, Beijing 100049, China; Institute of Biology, Leiden University, Sylviusweg 72, Leiden 2333 BE, The Netherlands

**Keywords:** behavioral barrier, birdsong dialects, phylogeography, playback experiment, speciation

## Abstract

Birdsong variation among populations within species has been regarded as a possible precursor for variation between species and may play a role in speciation. However, acoustic variation between 2 populations of the same or different species can have a variable impact on mutual responsiveness, and we currently lack sufficient insight into the underlying reasons. We report here on geographic song variation and responsiveness to playback for the light-vented bulbul (*Pycnonotus sinensis*), a species with recent range expansion in China. We recorded songs in 4 historical populations in the south and 6 newly established populations in the north. We tested responsiveness in 2 southern and 2 northern locations to songs from southern and northern birds. Besides songs from the other distribution range (south to north and north to south), we used recordings for playback from local, near-by, and far-away dialects from within their own distribution range. We confirmed distinct dialectal differentiation in the south and a more gradual pattern of geographic song divergence in the north. Birds in the south showed very little response to almost all nonlocal songs. In contrast, birds in the north showed a strong response to a wide-range of stimuli, just gradually fading from local, near-by, to far-away northern dialects, which may be related to song type sharing or individual mobility and aggression associated with the recent range expansion. We hereby add an asymmetric response pattern in responsiveness to the literature and review the current insights about the potential impact of song variation on avian speciation.

## Teaser

Songs of many songbirds vary geographically like human dialects and could play a role in reproductive separation of populations. We show that southern light-vented bulbuls have distinct dialects and responded nearly exclusively to anything else than their own dialect. Northern bulbuls are part of a recent range expansion, vary more gradually in song, and responded also to other dialects than their own. This asymmetric response may affect their chances of becoming separate species in future.

## Introduction

Song is an important sexual signal for mate attraction and territorial advertisement in many bird species and can play a crucial role in species recognition and speciation (Price 1998; Slabbekoorn and Smith 2002a). Birds recognize conspecific songs and discriminate against heterospecific songs based on detectable and consistent acoustic variation (Martin and Martin 2001; Hamao 2016). Even within the same species, populations in different regions usually exhibit variation in song, commonly referred to as dialects (Catchpole and Slater 1995; Podos and Warren 2007). Research on song nondiscrimination and discrimination among conspecific populations or among different subspecies have reported interesting but variable patterns, and our understanding of the underlying reasons is still far from complete.

Acoustically similar birds of the same population typically recognize and respond to each other. However, acoustically distinct birds from different populations do not necessarily discriminate against each other and the potential for a behavioral barrier to gene flow is not straightforward (Baker and Cunningham 1985; Slabbekoorn and Smith 2002b; Podos and Warren 2007). There are studies on playback tests that show discrimination against a dialect of a foreign subspecies, with stronger responses to local songs (Reichard 2014), but others report no discrimination against foreign dialects (Parra et al. 2017; Greig et al. 2021). These were studies with playbacks in a single population, but there are more elaborate studies showing both patterns (discrimination and nondiscrimination) in the same species. Mahoney et al. (2021) reported on 3 different subspecies of willow flycatchers (*Empidonax traillii*) in North-America (*brewsteri*, *adustus*, and *traillii*), which did not discriminate against each other, but all 3 exhibited a mutual discriminatory pattern with a fourth Mexican subspecies (*extimus*).

Response patterns between 2 different populations of birds with distinct song characteristics can be symmetrical (eg Lipshutz et al. 2017; Macedo et al. 2019), with both populations reacting to each other's songs (nondiscrimination) or both reacting stronger to their own than to the other's songs (discrimination). Response patterns can also be asymmetrical (eg Brooks and Wimberger 2023), with 1 population or subspecies recognizing the other, and 1 population discriminating. Such asymmetric response patterns have now been repeatedly found for parapatric conditions, across altitudinal and latitudinal gradients (Slabbekoorn and Smith 2002b; Colbeck et al. 2010; Kirschel et al. 2011; McEntee 2014). Several studies also tested both allopatric and parapatric conditions, which yielded the same consistent patterns for some studies (Dingle et al. 2010; Caro et al. 2013; Burbidge et al. 2015), and distinct patterns in others, where the asymmetric discrimination faded and was either replaced by mutual nondiscrimination (Jankowski et al. 2010) or mutual discrimination where birds actually met (Demko et al. 2019). We currently lack sufficient insight into the underlying reasons for these variable response patterns, and for a better understanding of the impact of acoustic variation among populations, we probably need a better understanding of the context and mechanism driving acoustic divergence among populations.

Geographic variation in advertisement song among bird species is the result of genetic and cultural processes, with a most prominent role for the latter in species that learn their songs (Williams and Slater 1990; Ellers and Slabbekoorn 2003), Genetically inherited morphology, physiology, and neurological templates, affect what sounds a bird can produce and to what extent they are selective or tolerant in tutor choice. This will typically result in increasing genetic and acoustic divergence with distance, for which the rate of geographic change will depend on dispersal tendency, and the copying accuracy and time window for song learning (Slabbekoorn and Smith 2002a; Podos and Warren 2007). However, especially post-dispersal learning may mean more shared songs among neighbors and more repertoire overlap within populations through convergence of locally interacting birds (Ellers and Slabbekoorn 2003), which could promote local nondiscrimination and discrimination of foreign songs (Vargas-Castro 2015). Seasonal migration on the other hand may yield spatial overlap for individual birds that breed far apart and the acoustic exposure to foreign signals, and may potentially reduce discrimination (Wright and Dorin 2001; Colbeck et al. 2010). Ecological selection pressures on acoustic structure, related to sound propagation or ambient noise profiles, may further shape geographic variation in bird songs through convergence for birds in the same habitat and divergence for birds in different habitat, yielding eco-typical song variation (Wiley and Richards 1978; Slabbekoorn and Smith 2002b; Tobias et al. 2010; Caro et al. 2013).

Light-vented bulbuls (*Pycnonotus sinensis*) in China provide an interesting case study of a species with strong dialectal variation (Yang and Lei 2008; Xing et al. 2013) and an interesting phylogeographic history after a recent range expansion (Wang et al. 2005; Xing et al. 2013). The species still has a near-endemic status to China, but was restricted to the southern half until the 1930s, and since then invaded the northern half of China (Williams 2000; Zhang et al. 2003; Fishpool and Tobias 2005). They still occurred at relatively low numbers in the northeast in the 1980s and 1990s. However, likely due to the steady increase in temperatures over the last decades and the species' ability to utilize human-created habitats, they have now become very common throughout their expanded range (Wen et al. 2014). Distinct and diversified song dialects were reported from the historical populations in the south (Jiang et al. 1996; Ding and Jiang 2005; Yang and Lei 2008). The northern populations contrast with the south in having a reduced syllable pool, which is shared over large distances, and in showing a clear pattern of greater divergence over larger distance in acoustic structure (Yang and Lei 2008; Xing et al. 2013). However, we have no insight yet into whether the overall geographic patterns in song dialects affect territorial responsiveness in either the historical distribution range of the south or the recent populations in the recently invaded range in the north.

In the current study, we report on variation in song structure and responsiveness among southern and northern populations of the light-vented bulbul in China, which represent historical and recently invaded distribution ranges, respectively. We recorded songs in 4 southern and 6 northern cities and used these to test responsiveness in 2 southern and 2 northern cities. We explored song similarity in acoustic structure among populations by comparing current measurements with genetic relatedness from a previous study. Furthermore, we conducted playback experiments to test response triggering potential of songs from populations of the same and the other part of the current distribution range (historical distribution or recent range expansion), using recordings from local birds, and birds from nearby and far-away localities. We expected stronger responsiveness to songs of more similar acoustic structure and sharing more dialectal song types. We also expected that genetic relatedness and dispersal history could reflect more recent experience with distant dialects especially in the northern populations, which may yield more nondiscrimination than discrimination.

## Materials and methods

### Study species

**Figure 1:**
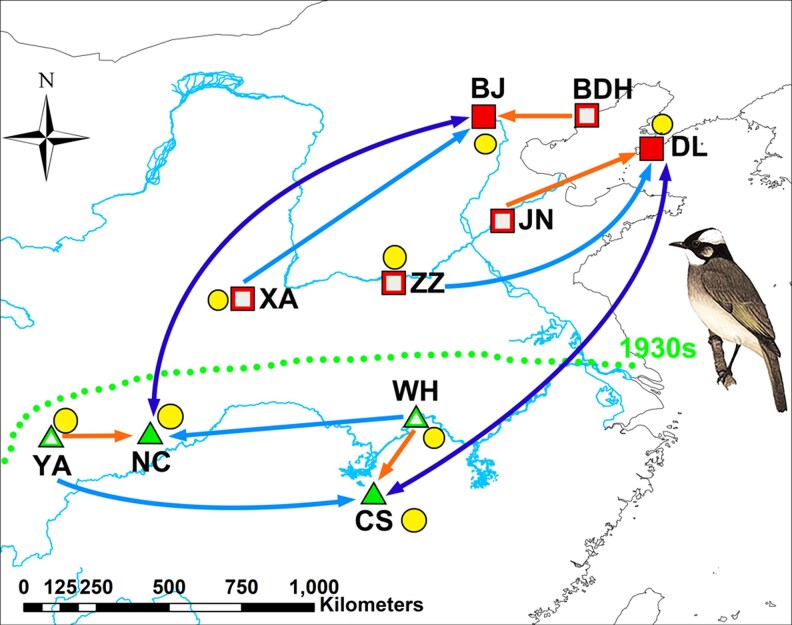
Map of populations and experimental design. Sample populations in the southern distribution range are indicated by triangles and sample populations in the northern distribution range by squares. The dotted line indicates the upper limit of the historic species distribution in the south and concerns the geographic line crossed after 1930 for a rapid range expansion into the north. Filled symbols indicate localities where we not only recorded songs, but where we also conducted playback experiments. Open symbols indicate localities where we only recorded songs. Arrows indicate the source of playback stimuli that we used in these 4 populations. In both the north and the south, we used song recordings from 1 near (orange) and 1 far (light-blue) population from within the same part of their distribution range (north or south), and from 1 population of the other region (dark blue). The yellow circles indicate genetic sampling localities. The full names of the sample populations, here labeled with abbreviations, are provided in Table S1.

The light-vented bulbul is a medium-sized (∼19 cm in body length and 40 g in body mass) oscine bird species of forest edges, scrubs, and urbanized environments, such as plantations, orchards, and urban parks (Williams 2000; Zhang et al. 2003; Fishpool and Tobias 2005) The species used to be a widespread resident south of the Yangtze River, China, in the Oriental region, and began expanding northward in the 1930s and has been spreading rapidly in the 1980s and 1990s. It now also occupies the wide Palearctic ecozone of northern China (Jiang et al. 1996; Ding and Jiang 2005; Wang et al. 2005). The vocal repertoire includes a single-syllable common call, an alarm call or mobbing call, a flight call, and songs that can exhibit dialectal variation in multisyllable song types, with a fixed sequence of syllables, each uttered once or a few times (Jiang et al. 1996; Ding and Jiang 2005; Yang and Lei 2008; Xing et al. 2013). We defined the populations in the south and near the Yangtze River as the historical, source populations, and the populations in the north as the newly colonized, range expansion populations (Fig. 1).

### Phylogeography

Although the general pattern of recent range expansion of the light-vented bulbul in China is clear (from the south to the north), we analyzed the genetic relatedness among populations with existing data. We retrieved partial fragment sequences of the mitochondrial cytochrome b (Cytb) and NADH dehydrogenase subunit 6 (ND6) genes (Song et al. 2013) for the geographic distribution of populations that we used for song recordings and playback in the current study. If we did not have a matching sampling site for the genetic data, we selected the nearest-by population. We were able to get sequence data from 8 relevant populations.

Sequences were aligned and geographically annotated in MEGA 7 (Tamura et al. 2011). The sequences of the 2 gene fragments were concatenated into a 1,305 bp long sequence for each individual. We calculated the pair-wise net genetic distance between populations with a maximum composite likelihood model, including both transition and transversion substitutions in MEGA 5. For some population pairs, the net genetic distance was just below zero, which we adjusted to 0, as this indicates no genetic differentiation. The data from the genetic distance matrix were used to generate a phylogenetic reconstruction via the neighbor-joining method. We used sequence data from a population from Taiwan as the outgroup. We used the clustering of populations based on genetic relatedness to compare the phylogenetic tree to an acoustic similarity-based phenetic tree. Furthermore, in order to assess gene flow among geographical populations, we calculated the Fst values between populations in DnaSP 5.0 (Librado and Rozas 2009), and subsequently converted the gene flow estimator Nm with the equation Nm = (1 − Fst)/2Fst.

### Song recordings

We recorded songs in 6 northern populations: Beijing (BJ), Dalian (DL), Beidaihe (BDH), Xian (XA), Zhengzhou (ZZ), and Jinan (JN), and 4 southern populations: Nanchong (NC), Changsha (CS), Yaan (YA), and Wuhan (WH) (see Fig. 1). Spontaneous songs (not triggered by playback) of 10 males in each population were recorded using a TASCAM DA-P1 recorder (Tascam, Japan) and a Sennheiser MKH 416 directional microphone (Sennheiser Electronic, Germany). All these songs were recorded during the early phase of the breeding season and were subsequently used to create playback stimuli (Fig. 1; Table S2). Playbacks can be used to test responsiveness to acoustic variation in a territorial or mate attraction context, and thereby test the potential impact on mutual exchange of reproductive individuals and gene flow (Slabbekoorn and Smith 2002a; Freeman and Montgomery 2017).

### Playback song stimuli

**Figure 2:**
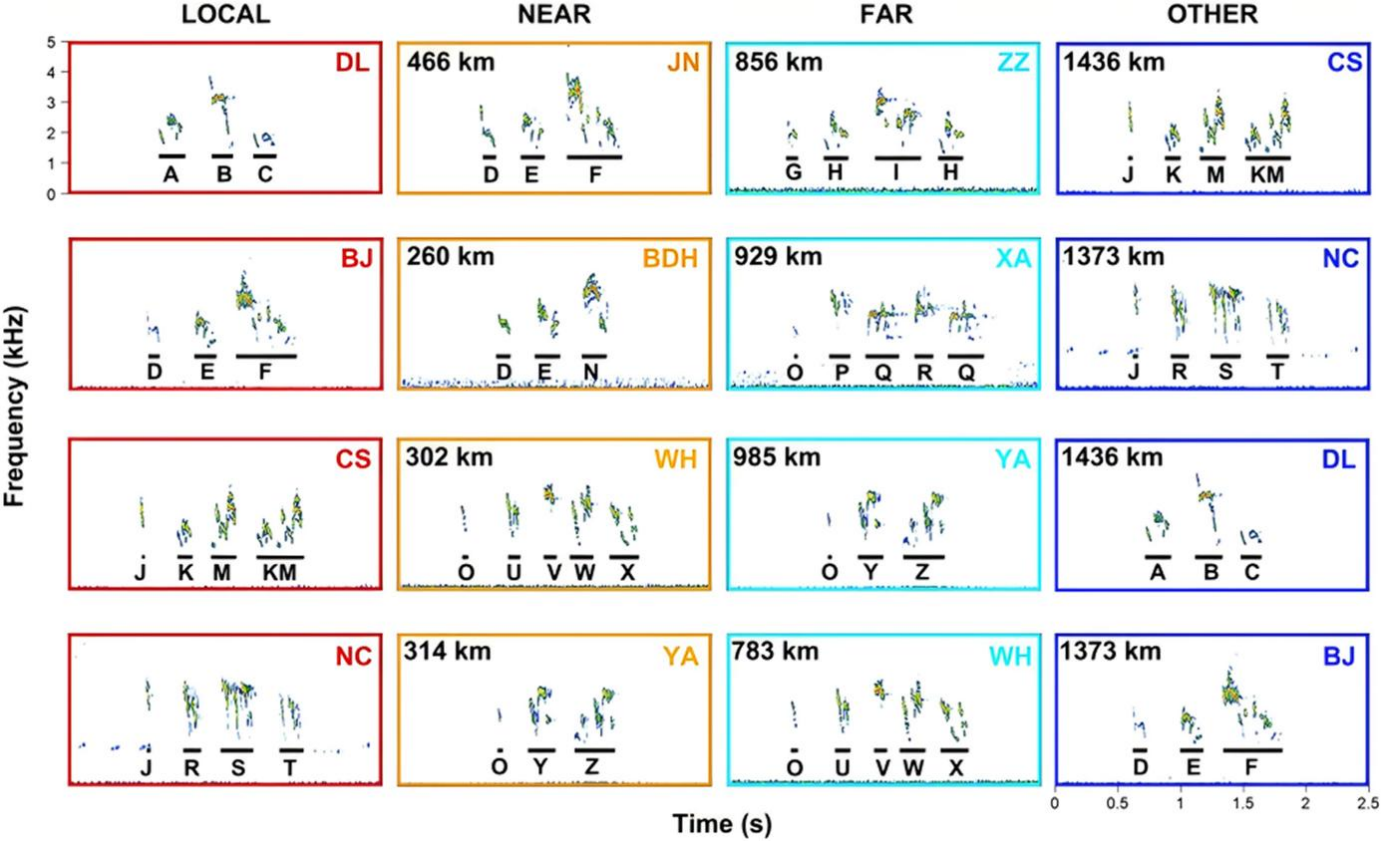
Sonographic overview of song variations among local populations and all other stimuli populations for playback. Each spectrogram is an example song used for playback as local, near, far, or other stimulus for the associated playback location. The letters below the syllables indicate different syllable types which reveal some of the variation in syllable sharing. The distances indicate the geographic separation between the local populations in which we conducted the playbacks to the source populations of each song stimulus in the same row.

We selected NC and CS for the southern populations and BJ and DL for the northern populations to test local responsiveness to geographic variation in song via playback experiments. We compared response strength to songs of the local population, a near-by and far-away population of the same region (historical source or recently expanded range), and a far-away population from the other region (Fig. 2). We used 1 high-quality recording of a clear song per male, with high signal-to-noise ratio, and without any prominent sound events in the background. Furthermore, all song recordings were band-pass filtered between 1 and 12 kHz, to eliminate any background noise outside the song frequency range. We repeated each song for 3 min to create playback files, using Avisoft-SAS Lab Pro v.4.52 (Avisoft Bioacoustics, Berlin, Germany). Intervals between songs were tailored to the natural singing behavior of light-vented bulbul.

We avoided playback of songs of their own and of near-by neighbors to exclude individual recognition and familiarity of birds to affect the response strength. The song of a particular male was used twice per playback population, which means that we used 4 sets (own, near-by, far-away, other) of 10 unique song stimuli from different males per population of origin (thus 40 unique songs) to test responsiveness of 20 unique males in 20 different territories per site (thus 80 playback trials per site). In this way, we reduced problems of pseudo-replication (Kroodsma 1989; Slabbekoorn 2013) through restricting the repeated use of the same stimuli in the same population more than twice.

We selected playback songs from the recorded samples that were typical for the local song dialect. The northern populations had fewer song types overall than the southern populations (7 vs 118), as reported in our previous study (Xing et al. 2013). The most common song types 1 and 2 are widespread in the north and shared among BJ, BDH, JN, and DL, while birds in DL also sing song type 3. Birds in ZZ and BJ sing song type 4; and XA harbors 3 unique song types 5, 6 and 7 (see Fig. 1 in Xing et al. 2013). All these 7 song types were included in the playback song stimuli of the northern populations. The southern populations exhibit quite a different pattern, where each population has their own dialect, each with several unique song types.

### Playback procedure

We conducted playback experiments from 9 April to 30 May during the early breeding seasons in both southern and northern populations. We selected singing male bulbuls that were paired (near-by female observed) as target males for playback. We assessed behavioral and singing responses during and after playback periods. Noisy places were avoided and we also skipped territories with near-by neighbors or other loud bird species actively singing. We chose a place for speaker placement in the center of the territory, based on observations of multiple perches used for singing. We placed the speaker always close to trees branches or electric wires to provide sufficient perch options for bulbuls and to guarantee spatial resolution to score approach responses. We used a Samsung S7 edge smartphone connected with Bluetooth to a JBL Clip + Portable Bluetooth Speaker (JBL, Los Angeles, USA). The speaker was attached to a wooden stick and positioned 1.5 m above the ground, which is a typical song post height in this species. The distance between the speaker and focal bird was up to 10 m. One researcher observed and orally narrated the behavioral response of males during playback, while another researcher recorded the vocal response of the target bird. Both recording tracks were made with a TASCAM DA-P1 recorder (Tascam, Japan) and a Sennheiser MKH 416 directional microphone (Sennheiser Electronic, Germany).

**Figure 3:**
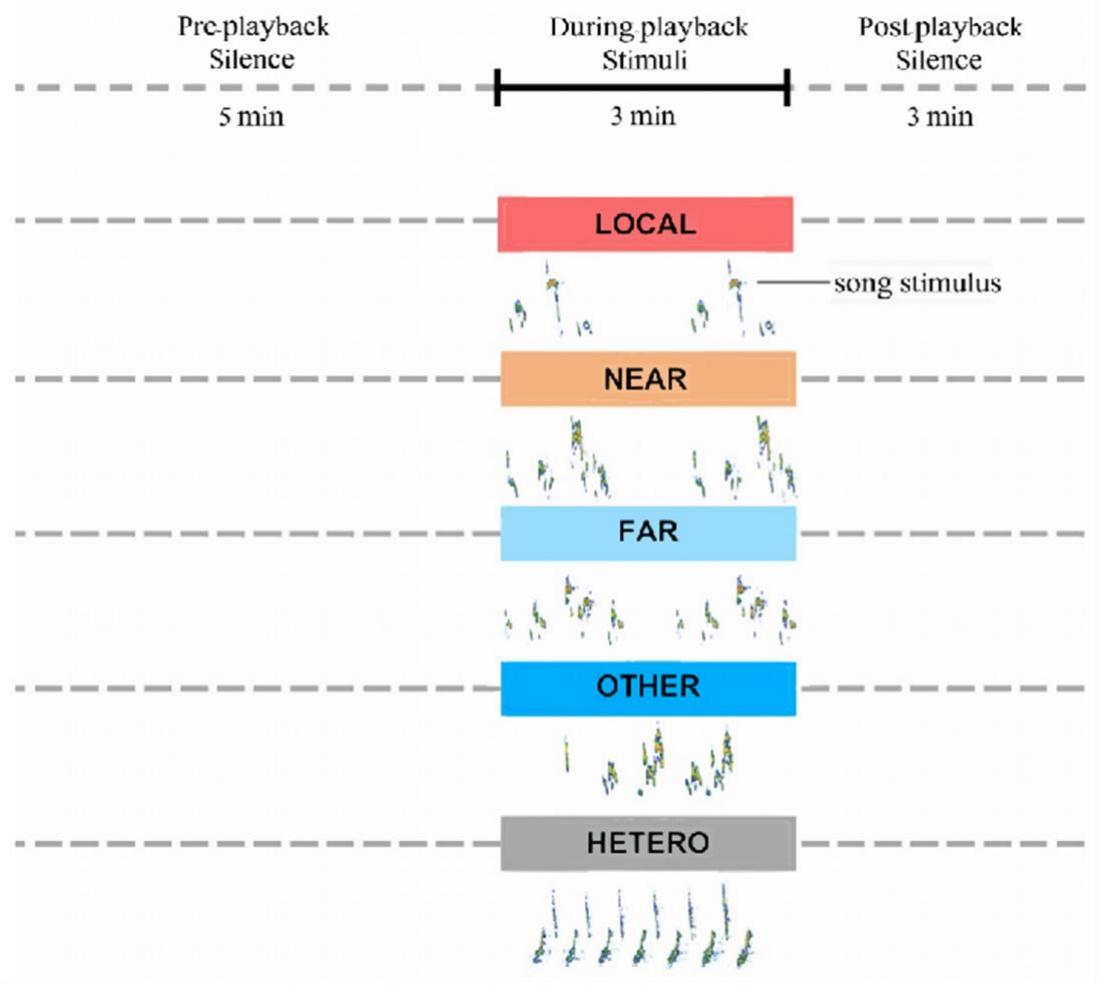
Schematic representation of the time line for observations and recordings during a playback trial with the exposure to songs of 3 min and a preplayback period of 5 min and a post-playback period of 3 min. Examples of conspecific and hetero-specific songs are provided in the spectrograms below. For playback procedures, see “Playback song stimuli”.

We started each series of playback with heterospecific great tit (*Parus major*) song to get an experimental control of baseline behavior during playback set-up, sound playback, and observer presence. The great tits are sympatric and co-occur in the same habitat as light-vented bulbuls. Both species are not ecological competitors, have both loud but structurally very different songs and they are not known or expected to respond to each other's vocal presence. After the great tit song control, we played the 4 bulbul song treatments of different origin in random order. We therefore had a playback sequence of 5 different song stimuli, with each song played 5 times for 11 min, resulting in total trial duration of 55 min per bird territory. Every playback trial consisted of 3 periods per song stimulus (Fig. 3): preplayback (5 min, the time before or in between subsequent playbacks), during-playback (3 min), post-playback (3 min). The heterospecific control never elicited any obvious response and the quantification of the relatively low-level of excitement during this playback period thereby adequately served as baseline behavior for the evaluation of any responsiveness during the following bulbul song stimuli. Typically, the birds also recovered to this behavioral state (eg, preening, etc.) during the interval between 2 playbacks within a trial of 5.

### Behavioral response measurements

During playback, responsive bulbuls performed flights towards and over the speaker, with increased rate of head turning, and raised attention for the speaker area, which we interpreted as searching for the speaker location as the source of the song of an apparent territorial intruder. The birds often approached the speaker, in multiple flights among different perches, and sometimes by small jumps on their perches, while producing calls or songs. Behavioral response variables were scored for the during- and post-playback periods and included: (i) search for speaker (SS), which was a categorical score of whether the focal bird turned his head and/or changed position towards the speaker: Yes = 1, No = 0; (ii) latency to first approach (LA, s), which was defined as the time from stimulus onset to initial approach; (iii) duration of response (DR, s), which concerned the time passed between initial response and when the bird was not visible or audible anymore, or when it was seen more than 20 m away from the speaker, preening, foraging, or singing to neighbors; (iv) approach distance (AD), which was measured as the minimum distance of the focal bird to the speaker, in the following categories: 0 ≤ 1 m, 1 = 1–5 m, 2 = 5–10 m, 3 ≥ 10 m; (v) number of perch changes (NOC), calculated as the number of position switched in tree branches or on electric wires; (vi) latency to first song (LS, s), which was defined as the time from stimulus onset to the start of singing; (vii) number of response songs (NOS); (viii) mean song length (LM, s), calculated as (total song length)/(NOS); (ix) total song length (LT, s), calculated as total length of all songs the focal birds produced.

### Song analyses

We measured and compared the songs recorded in the first preplayback period and responsive songs sung during and after the playbacks as part of the integrated measure of behavioral responsiveness. We recorded 5 min of spontaneously sung songs (not triggered by playback) for each male in the first preplayback period of each trial. Song recordings were converted into WAV format and were digitized at a sampling rate of 22.05 kHz, 16-bit resolution. We selected high-quality songs for analysis with high signal-to-noise ratio and low background noise. We visualized recordings for measurements with standardized settings for sonograms (Hamming window, FFT size = 256, frame size = 100%, overlap = 50%, frequency resolution = 86 Hz, temporal resolution = 5.8 ms) using Avisoft-SAS Lab Pro v.4.52 (Avisoft Bioacoustics, Berlin, Germany).

We measured the following song variables including: (i) song duration (D, s), total length of the target song (time of song ending − time of song beginning); (ii) maximum frequency (*F*_max_, kHz), highest trace of sound attributed to the target song; (iii) minimum frequency (*F*_min_, kHz), lowest trace of sound attributed to the target song; (iv) bandwidth (*F_b_*, kHz), ie frequency range (maximum frequency − minimum frequency); (v) peak frequency (*F*_peak_, kHz), the frequency of maximum energy in the target song; (vi) number of syllables per song (NS), counted by the number of syllable onsets in the target song; (vii) syllable rate (RS, number/s), which was calculated as the number of syllables per song/the song duration; (viii) number of unique syllable types per song (NUS), calculated as the number of unique syllable type onsets in the target song (c.f. Xing et al. 2013).

All songs were recorded during spontaneous singing bouts, without using playback to trigger vocal activity. Song recordings of high-quality were selected from 10 northern and southern populations to analyze geographic song variation among the populations (Table S2). Six acoustic parameters were measured and analyzed (definitions see above): maximum frequency (*F*_max_, kHz), minimum frequency (*F*_min_, kHz), peak frequency (*F*_peak_, kHz), song duration (D, s), number of syllables per song (NS), and syllable rate (RS, number/s).

### Statistical analyses

To reduce our set of acoustic measurements on song variation into a few axes, we performed a principal component analysis (PCA) using the “psych” software package (R package version 2.0.9, https://CRAN.R-project.org/package=psych) (Revelle 2024). A hierarchical cluster analysis was used, with an average linkage method (UPGMA), based on the dissimilarity matrix of the Euclidean distances (Slabbekoorn et al. 1999; Romesburg 2004). The degree of similarity among bulbul songs is thereby represented by the proximity of the horizontal branches in the phenetic tree with the most similar songs closest to one another. We used this clustering based on acoustic similarity to compare to the phylogenetic tree based on genetic clustering, reconstructed with data from a previous study (Song et al. 2013). Furthermore, we plotted population averages for the first 2 principal components to visualize variation in acoustic structure among populations in our current measurements to compare to the dialectal variation in song type variation expressed as the Jaccard's coefficient in pair-wise comparisons among southern and northern populations, as reported in our previous study (Xing et al. 2013). In that study, the Jaccard's coefficient was calculated as the number of shared song types divided by the total number of different song types in both populations together. We conducted a PCA on the acoustic variables of the 4 playback populations, depicting the PC1 and PC2 acoustic variations in the song stimuli used for playback. This analysis allowed detailed inspection of acoustic similarity as the potential cause of variation in responsiveness to songs from different source populations.

To integrate our set of behavioral response measurements into a single measure of overall response strength, we again performed a PCA using the “psych” software package (R package version 2.0.9, https://CRAN.R-project.org/package=psych). We used the Kaiser–Meyer–Olkin test (KMO, “psych” package in R) to assess the suitability of our measurements to be included in the PCA. The sampling adequacy measure was above 0.7 for all response variables and thereby considered suitable for the PCA. The response variables for each population were also sufficient for data reduction according to Bartlett's test of sphericity (*P* values < 0.001 for all data; “Bartlett. Test” function, “psych” package in R).

We extracted 2 principal components (PC1 and PC2) with eigenvalues above 1, which together explained up to 70% of the total variance for each population. PC1 showed high positive loadings for variables related to vocal activity and response duration (DR, NOC, NOS, LM, LT), and negative loadings for approach measures (LA, AD, and LS). Therefore, PC1 represented the overall response intensity or aggressive engagement toward playback stimuli, with higher scores indicating stronger and more persistent responses. PC2, which explained an additional 18% to 21% of the variance, had loadings that contrasted vocal behaviors (NOS and LT) with spatial behaviors (AD and NOC), suggesting that PC2 reflected variation in response type rather than response strength. We performed a Kruskal–Wallis rank sum test to compare the response strength (for PC1 and PC2) to the different stimuli in during- and post-playback periods respectively, and we conducted post hoc analyses (Wilcoxon rank sum tests) for multiple comparisons of response strength among the 5 song stimulus categories, using “wmc” function in R (http://www.statmethods.net/RiA/wmc.txt). Only PC1 was able and sufficient to show significant variation among different stimuli (explaining 59.8% to 68.4% of the variance during playback and 55.9% to72.3% in the post-playback period). PC2 was therefore left out of further analyses (explaining an additional 18.3% to 21.4% during playback 14.8% to 18.5% and in the post-playback period).

Subsequently, we conducted linear mixed models (LMM) on PC1 to test the difference of behavioral responses of light-vented bulbuls from each population in 2 periods respectively (using the package “lme4”, R package version 1.1-27.1, https://CRAN.R-project.org/package=lme4). We included the behavioral playback response (PC1) as the response variable, stimulus category, and the order of stimulus presentation as fixed effects, and individual identity (indID) as random effect. Subsequently, we used Tukey's test for post-hoc comparisons (using the package “multcomp”, R package version 1.4-17, https://CRAN.R-project.org/package=multcomp). We conducted all statistical analyses in R v.4.0.5 (R Development Core Team, http://www.r-project.org) and RStudio v.1.3.1093, and figures were created using the “ggplot2” package in R (R package version 3.3.2, https://CRAN.R-project.org/package=ggplot2).

## Results

### Genetic relatedness and song similarity

**Figure 4:**
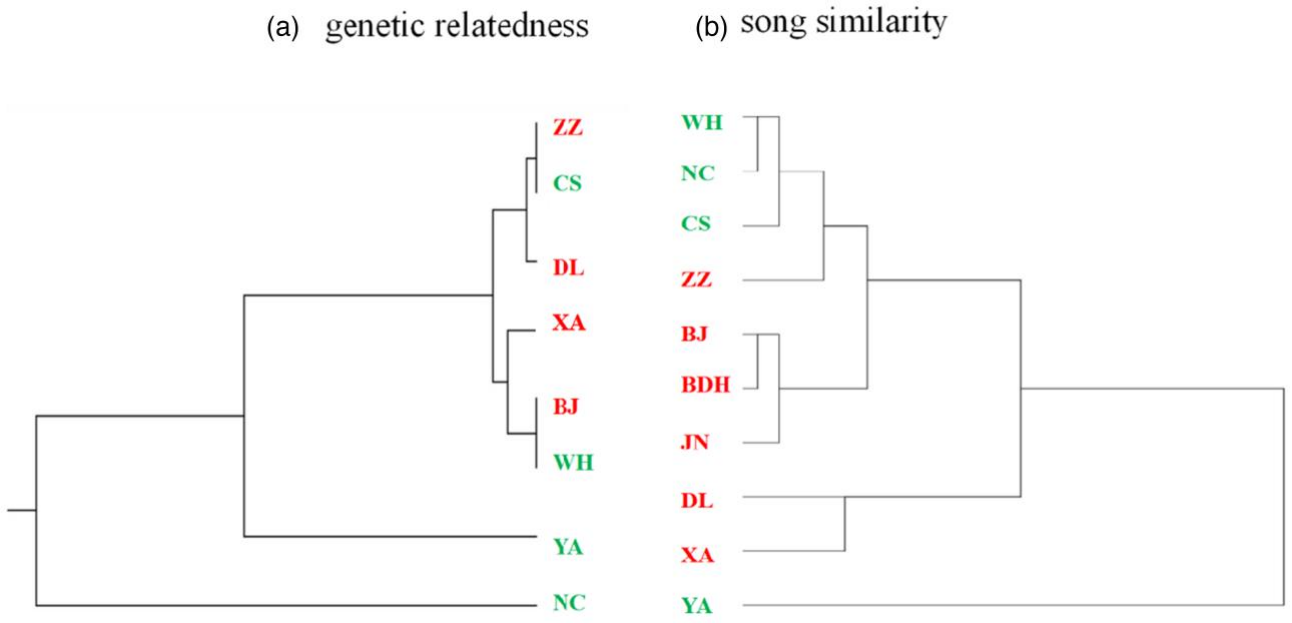
Cluster results based on (a) genetic distance and (b) song similarity among different populations. See Fig. 1 for population abbreviations.

Our phylogenetic analysis on 8 of our populations for which we had sequence data revealed no clear divergent pattern between northern and southern populations (Fig. 4a), and the Fst values among population pairs were very low (Table S3). The estimated Nm between geographic populations were all larger than 1, indicating considerable gene flow among populations, the Nm values are especially high among the newly established populations (eg DL/BJ: 95.6, Changan (CA)/BJ: 42.5) (Table S4). Despite gene flow among all populations, the southern populations were genetically more differentiated, while the northern populations seem to have originated from different populations in the south.

The phenetic analyses on acoustic variation (based on 683 spontaneously sung song in the preplayback period) showed a contrasting pattern in having a clear clustering of southern and northern populations (Fig. 4b), with an intermediate position for ZZ. However, in the southern and northern populations, 3 populations diverged from their expected cluster: YA does not cluster close to the other southern populations NC, WH and CS, DL does not cluster close to the other northern populations BJ, BDH, JN, while XA does not cluster close to ZZ. The lack of east–west concordance in song similarity does agree with the east–west segregation in genetic relatedness. YA and NC are geographically proximate (closer to each other than the northern populations are in the acoustically similar cluster of BJ, BDH, and JN), yet they occupy distinct acoustic positions, a pattern that may reflect cultural drift in vocalizations.

### Responsiveness to playback

**Figure 5:**
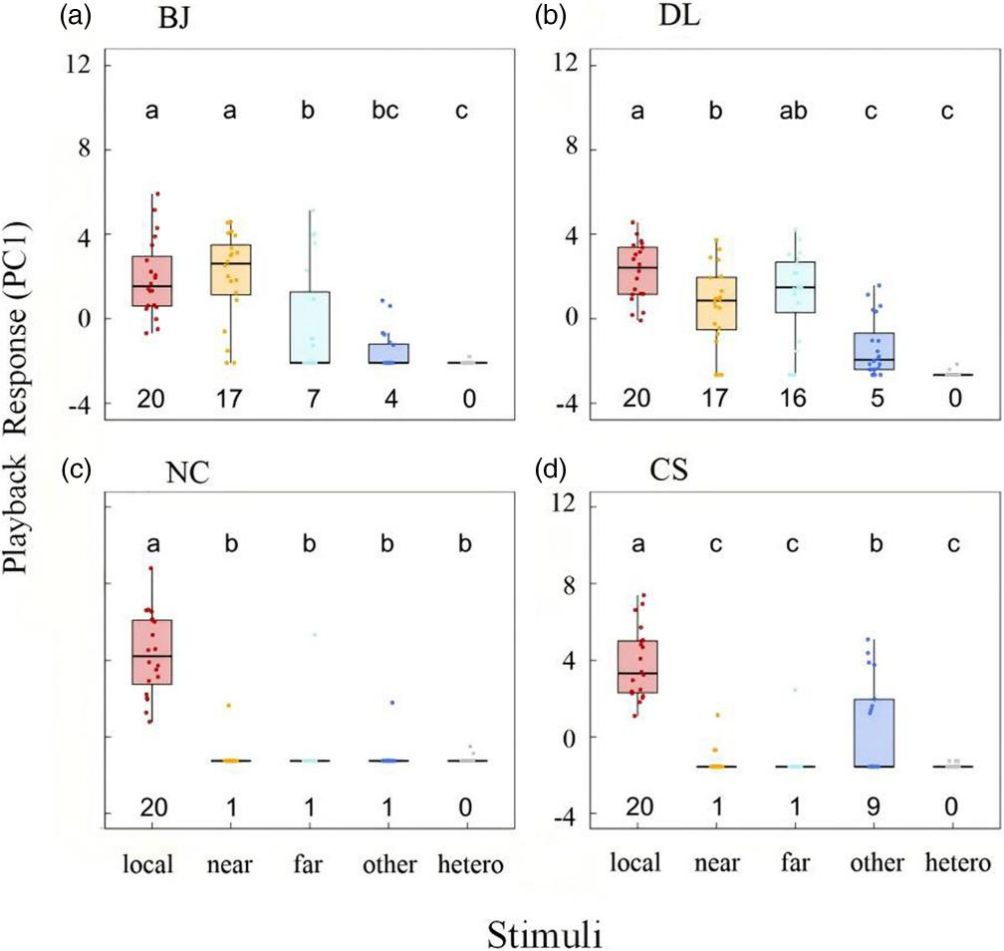
Responses of territorial males in the 2 northern (a—Beijing [BJ]; b—Dalian [DL]) and the 2 southern (c—Nanchong [NC]; d—Changsha [CS]) populations to multistimuli playback experiments. The number under each box plot represents the number of individuals that responded to the playback.

In 80 playback experiments, all males responded to songs of their own populations, in both southern and northern populations (Fig. 5). They did not respond equally strongly to songs from populations that were not their own and there was a clear distinction between the patterns for the southern and northern populations. Higher values of PC1 reflected more searching for speaker, longer response duration, more perch changes, more songs sung in response, and shorter AD, faster approach and faster singing response. There was an overall asymmetric pattern in responsiveness, as expressed by PC1: the birds in the 2 southern populations responded to songs of their own population, and discriminated against all others, except for the songs of the northern population Dalian (DL) in the southern population of Changsha (CS), while the birds in the 2 northern populations showed a general gradual decline in responsiveness from songs of their own populations, to songs from the near, far, other, and hetero-specific song categories.

Males from Beijing (BJ) responded to local songs equally as to those from the near-by site, but significantly less to those from far-away and other (Fig. 5a). Males from Dalian (DL) responded significantly less to near-by, and other, compared with local, but the response to near-by and far-away was again significantly stronger than to other. The order of song stimulus presentation had no significant effect and dropped out for the final models (LMM: during-playback: CS: *F*_1,100_ = 3.478, *P* = 0.065; NC: *F*_1,80_ = 2.067, *P* = 0.154; BJ: *F*_1,80_ = 0.611, *P* = 0.437; DL: *F*_1,80_ = 0.094, *P* = 0.760). Responses and statistical results for the post-playback period were similar to those for the during-playback period. Detailed results of acoustic and behavioral responses are provided in Table S5-S9.

## Discussion

Our results on song similarity among populations revealed a crude match with geographic distribution: southern populations clustered together and so did northern populations. However, the separation was not perfect, with some populations deviating from their expected cluster. The acoustic and genetic data also did not match, as there was no clear divergence between northern and southern populations in terms of genetic relatedness, and there only was some east–west segregation in clusters of populations. In terms of response triggering potential of songs, we found a clear asymmetric pattern in response strength, with strong discrimination in the historical distribution of the southern populations and a gradual decline in responsiveness and weaker discrimination in the northern populations. Neither similarity in acoustic song structure among populations nor genetic relatedness can explain the distinct asymmetric response pattern to playback between the historic distribution in the south and the recent range expansion in the north. We argue below that the asymmetric variation in responsiveness may be related to range expansion dependent dialectal variation of song and syllable types and/or to dispersal-related response tendency.

### Song variation and territorial responsiveness

Discrimination of nonlocal song variation is seen as a prerequisite for reproductive isolation when populations come into secondary contact or for a promoting role in speciation of divergent populations in general (Baker and Cunningham 1985; Price 1998; Slabbekoorn and Smith 2002a; Podos and Warren 2007). In the southern populations, we found strong dialects and clear discrimination against each other's songs, even when populations were geographically close. This is in line with a role of intra-specific acoustic divergence in promoting speciation. In contrast, in the northern populations, we also found song divergence, but less discrimination with responses gradually decreasing as geographic distance between populations increases.

The pattern of geographic variation in responsiveness of the light-vented bulbuls could be determined by song and syllable types or acoustic structure, which varied relatively independently in our data. Song type sharing in the north reflected the track of recent range expansion better than acoustic structure and we argue is the most likely explanation for why both northern populations responded to a larger range of song stimuli than did the 2 southern populations. The northern sites share similar song types (Xing et al. 2013), which is only partly reflected in our current analysis of the acoustic structure. There are 3 widespread song dialectal areas: one most prominent dialect is reported for Beijing (BJ), Beidaihe (BDH), Jinan (JN), and Dalian (DL) (all 4 populations sharing the most common song types), and 2 other dialects (with different song types), found in Xian (XA) and Zhengzhou (ZZ) (Xing et al. 2013). This matches with the decline in responsiveness in the northern populations to the song stimuli from near to distant populations.

However, neither song type sharing nor acoustic structure cannot explain why birds from Dalian (DL) responded to songs from Zhengzhou (ZZ) as strong as to local songs. Furthermore, the birds of the southern Changsha (CS) population responded significantly less strong to the songs of the northern Dalian (DL) population compared with local songs, but there was still a considerable response with 9 out of 20 birds responding to this far-away other population. Interestingly, despite the lack of acoustic similarity, there is a relatively close relationship genetically between Changsha (CS) in the south and Dalian (DL) in the north. Whether the patterns of nondiscrimination and discrimination will affect or correlate to gene flow among populations may depend on whether dispersing males can establish a territory and whether dispersing females are as or more discriminatory than the males (Price 1998; Slabbekoorn and Smith 2002a).

### Dispersal tendency and learned responsiveness

Discrimination of nonlocal songs may promote speciation, but is not always a straightforward correlate to acoustic divergence among populations. The recent range expansion towards the north into another ecological zone may add a factor promoting speciation potential, as song variation may become a cue for local adaptation to southern or northern environmental conditions (Slabbekoorn and Smith 2002a). Furthermore, behavioral syndromes that facilitate successful dispersal may also influence geographic variation in both song divergence and behavioral responsiveness, and thereby affect gene flow (see eg Koolhaas et al. 1999; Sih et al. 2004). Individual dispersal syndromes tend to be different between populations that are historically old or newly established, with stronger dispersal tendencies being correlated with higher aggression levels in the newly established populations (Duckworth and Badyaev 2007), which raises potential for the co-evolution of these traits during range expansions (Canestrelli et al. 2016). Consequently, the less restricted discrimination patterns of light-vented bulbuls in the northern populations of our study may be the result of relatively high song type sharing among populations, but may also be determined by highly mobile and aggressive birds in the newly invaded distribution range. And these factors are not mutually exclusive.

Moreover, more dynamic spatial mixing during range expansion may increase the likelihood of exposure to dialectal variation of different areas. This could have similar effects as seasonal overlap among migratory and sedentary populations of the same species. For example, in black-throated blue warblers (*Setophaga caerulescens*), acoustic exposure during migration may allow birds in southern populations to hear and potentially learn songs on their breeding grounds from seasonally visiting birds of northern populations. Southern birds may therefore show no discrimination between local and foreign dialects, whereas the migratory birds of northern populations lack such exposure on their breeding grounds and may therefore not exhibit the same response pattern and discrimination instead (Colbeck et al. 2010).

Furthermore, several studies have tested both allopatric and parapatric conditions, which revealed differences between birds that vary in auditory experience with the other song variant. For example, Jankowski et al. (2010) found that the 2 species of *Henichorina* woodwrens showed mutual discrimination of each other's songs, and strong aggressive responses in replacement zones. However, as the distance from replacement zones increased, individuals showed reduced aggression toward heterospecific songs, suggesting that their song discrimination may depend on individual experience and a learned component. A shaped by auditory experience may similarly apply to our case in the northern populations of the light-vented bulbuls. During their range expansion, these populations may have been exposed to and accumulated a diversity of regional song variants, which could have influenced song learning processes and consequently reduced their ability to discriminate among songs. Such effects may, in turn, potentially influence gene flow and speciation (Irwin and Price 1999; den Hartog et al. 2008; Lipshutz et al. 2017).

### Asymmetric responsiveness and speciation

The overall pattern of playback results in our study adds a clear case of asymmetric responsiveness in a novel ecological context. Asymmetric response patterns have actually been found in quite a few more studies. Little greenbuls (*Andropadus virens*) in Cameroon and Uganda also responded asymmetrically (Slabbekoorn and Smith 2002a, 2002b; Kirschel et al. 2011), with rainforest birds in the south of both countries discriminating, and ecotone birds in the north in both countries not. McEntee (2014) found asymmetry in the heterospecific response patterns between 2 morphologically very similar sunbirds, *Nectarinia fuelleborni* and *N moreaui*. The latter species was the only 1 of the 2 discriminating against the other in parapatry.

While asymmetric response patterns of nondiscrimination and discrimination are likely to affect reproductive exchange between adjacent populations (Lipshutz et al. 2017; Hsiao et al. 2021). For example, Halfwerk et al. (2016) further demonstrated asymmetrical gene flow in the contact zone between these 2 subspecies, with a greater frequency of *H. l. leucophrys* alleles appearing in *H. l. hilaris* populations, consistent with the previously observed asymmetrical responses to playback.

In addition to asymmetric patterns of discrimination and nondiscrimination, symmetric nondiscriminatory (eg Parra et al. 2017; Greig et al. 2021) and symmetric discriminatory response patterns (eg Irwin et al. 2001; Mahoney et al. 2021), have been shown to influence gene flow among populations. More studies are needed in which both causes and consequences of acoustic divergence and behavioral responsiveness are studied at individual and population level.

## Conclusions

Our study adds another interesting case to the literature with respect to geographic variation in song divergence and behavioral responsiveness. The well-documented recent range expansion and the associated phylogenetic data provide an ecologically and evolutionary novel context for asymmetrical responsiveness among populations of different parts of the species distribution range. The distinct asymmetry in responsiveness between the northern and southern populations of light-vented bulbuls may be explained by either or both: (i) dialectal variation of song types, not necessarily reflected in divergence of acoustic structure, and (ii) invasion-related bird mobility, with birds of a more aggressive dispersal syndrome in the north responding less discriminatory than birds in the south. Independent of the causes, we also still need more insight into the potential consequences of asymmetric response patterns for biased gene flow and speciation. We believe this requires more integrated field studies on geographic variation in song, territorial responsiveness, and observations on acoustic mate choice or genetic data on hybridization.

## Supplementary Material

arag012_Supplementary_Data

## Data Availability

The data underlying this article are available in the Dryad Digital Repository, at https://doi.org/10.5061/dryad.k3j9kd5pj.
