# Supplementary material for: Asymmetry in responsiveness to playback of geographic song variation after a recent range expansion in light-vented bulbuls
Source: Behav Ecol. 2026 Feb 10;37(2):arag012. doi: 10.1093/beheco/arag012 (PMC13017478; doi:10.1093/beheco/arag012)
Supplement: arag012_Supplementary_Data [file arag012_supplementary_data.docx]

**Supplementary materials**

**Table S1:** Information on recording localities and years of light-vented bulbul song stimuli used in the playback experiments.

| **Stimuli** | **Locality** | **Coordinates** | **Recording year** | **Individuals** |
| --- | --- | --- | --- | --- |
| **Colonial population** | | | | |
| DL | Dalian, China | 38.91°N, 121.61°E | 2016, 2018 | 20 |
| BJ | Beijing, China | 39.90°N, 116.41°E | 2018 | 10 |
| BDH | Beidaihe, China | 39.94°N, 119.60°E | 2010 | 10 |
| JN | Jinan, China | 36.67°N, 116.99°E | 2010 | 10 |
| ZZ | Zhengzhou, China | 34.75°N, 113.62°E | 2011 | 10 |
| XA | Xian, China | 34.26°N, 108.94°E | 2006 | 10 |
| **Source population** | | | | |
| NC | Nanchong, China | 30.08°N, 106.08°E | 2018 | 10 |
| YA | Yaan, China | 29.98°N, 103.01°E | 2006 | 10 |
| WH | Wuhan, China | 30.59°N, 114.31°E | 2016 | 10 |
| CS | Changsha, China | 28.21°N, 113.00°E | 2016, 2018 | 20 |

Stimuli are indicated by abbreviations of recording localities.

**Table S2:** Information of the natural song recordings of Light-vented bulbuls.

| **Population** | **North/South Population** | **City** | **Abbreviation of the City names** | **Number of individuals analyzed** |
| --- | --- | --- | --- | --- |
| 1 | N | Dalian | DL | 20 |
| 2 | N | Beijing | BJ | 19 |
| 3 | N | Beidaihe | BDH | 18 |
| 4 | N | Jinan | JN | 20 |
| 5 | N | Xian | XA | 8 |
| 6 | N | Zhengzhou | ZZ | 12 |
| 7 | S | Yaan | YA | 10 |
| 8 | S | Wuhan | WH | 20 |
| 9 | S | Nanchong | NC | 28 |
| 10 | S | Changsha | CS | 15 |
| Total |  |  |  | 170 |

**Table S3:** Fst values among populations based on mtDNA sequences

| **Pop ID** | **Dalian** | **Beijng** | **Jiaozuo** | **Changan** | **Huangshi** | **Yichun** |
| --- | --- | --- | --- | --- | --- | --- |
| #Dalian | — | 0.0052 | 0.0328 | 0.0386 | 0.0542 | NA |
| #Beijng |  | — | 0.0648 | 0.0117 | NA | 0.0826 |
| #Jiaozuo |  |  | — | 0.0773 | 0.0700 | NA |
| #Changan |  |  |  | — | 0.0616 | 0.0385 |
| #Huangshi |  |  |  |  | — | 0.1163 |
| #Yichun |  |  |  |  |  | — |

The population of only one individual was excluded.

NA, The Fst valued was estimated in negative, possibly because the larger within population variance than that between the populations, while the Fst value is define between 0-1

**Table S4:** Nm values between populations

| **Pop ID** | **Dalian** | **Beijng** | **Jiaozuo** | **Changan** | **Huangshi** | **Yichun** |
| --- | --- | --- | --- | --- | --- | --- |
| #Dalian | — | 95.654 | 14.744 | 12.454 | 8.725 | NA |
| #Beijng |  | — | 7.216049 | 42.23504 | NA | 5.553269 |
| #Jiaozuo |  |  | — | 5.968305 | 6.642857 | NA |
| #Changan |  |  |  | — | 7.616883 | 12.48701 |
| #Huangshi |  |  |  |  | — | 3.799226 |
| #Yichun |  |  |  |  |  | — |

The gene flow was estimated by Nm with the equation Nm = (1-Fst)/2Fst

NA, the Nm value could not be calculated due to the negative Fst value

**Table S5:** Song variables of natural songs of males in DL, BJ, CS and NC. Number in parentheses indicates the number of males we

analyzed. Means are shown ± SD.

| **Population** | **D (s)** | **F_max_ (kHz)** | **F_min_ (kHz)** | **F_b_ (kHz)** | **F_peak_ (kHz)** | **NS** | **RS (number/s)** | **NUS** |
| --- | --- | --- | --- | --- | --- | --- | --- | --- |
| DL | 1.232 ± 0.306  (*N* = 14) | 3.606 ± 0.229  (*N* = 14) | 1.468 ± 0.145  (*N* = 14) | 2.139 ± 0.247  (*N* = 14) | 2.978 ± 0.234  (*N* = 14) | 4.765 ± 1.394  (*N* = 14) | 3.859 ± 0.634  (*N* = 14) | 3.971 ± 1.371  (*N* = 14) |
| BJ | 1.135 ± 0.253  (*N* = 11) | 3.885 ± 0.180  (*N* = 11) | 1.547 ± 0.174  (*N* = 11) | 2.339 ± 0.230  (*N* = 11) | 3.135 ± 0.309  (*N* = 11) | 4.000 ± 0.625  (*N* = 11) | 3.567 ± 0.451  (*N* = 11) | 3.619 ± 0.854  (*N* = 11) |
| CS | 1.302 ± 0.270  (*N* = 15) | 3.560 ± 0.225  (*N* = 15) | 1.328 ± 0.090  (*N* = 15) | 2.232 ± 0.245  (*N* = 15) | 2.725 ± 0.243  (*N* = 15) | 4.881 ± 0.962  (*N* = 15) | 3.782 ± 0.404  (*N* = 15) | 4.269 ± 0.914  (*N* = 15) |
| NC | 1.113 ± 0.178  (*N* = 8) | 3.644 ± 0.187  (*N* = 8) | 1.440 ± 0.158  (*N* = 8) | 2.205 ± 0.217  (*N* = 8) | 3.044 ± 0.254  (*N* = 8) | 4.088 ± 0.900  (*N* = 8) | 3.679 ± 0.423  (*N* = 8) | 3.912 ± 0.514  (*N* = 8) |

**Table S6:** Kruskal-Wallis rank sum test and multiple comparisons (Wilcoxon rank sum tests) of 5 song variables that were not follow normal distribution for natural songs of males in DL, BJ, CS and NC. Probability adjustment = “ holm ”.

|  | **D** | | **F_min_** | | **F_b_** | | **NS** | | **NUS** | |
| --- | --- | --- | --- | --- | --- | --- | --- | --- | --- | --- |
| *H* | 20.507 | | 54.301 | | 18.34 | | 32.183 | | 11.674 | |
| df | 3 | | 3 | | 3 | | 3 | | 3 | |
| *P* | **< 0.001***** | | **< 0.001***** | | **< 0.001***** | | **< 0.001***** | | **0.009**** | |
| ***Multiple comparisons (Wilcoxon rank sum tests)*** | | | | | | | | | | |
|  | ***P*** | ***T*** | ***P*** | ***T*** | ***P*** | ***T*** | ***P*** | ***T*** | ***P*** | ***T*** |
| BJ - NC | 0.477 | 645.5 | **0.019*** | 452.0 | **0.032*** | 452.5 | 0.973 | 717.0 | 0.619 | 626.0 |
| BJ - DL | 0.187 | 1104.5 | **0.044*** | 1056.0 | **< 0.001***** | 748.0 | **0.004**** | 912.0 | 0.173 | 1117.0 |
| BJ - CS | **< 0.001***** | 783.0 | **< 0.001***** | 359.0 | 0.101 | 1047.0 | **< 0.001***** | 622.0 | **0.006**** | 912.0 |
| NC - DL | 0.242 | 937.0 | 0.330 | 1018.5 | 0.438 | 982.5 | **0.018*** | 782.0 | 0.619 | 1281.0 |
| NC - CS | **< 0.001***** | 613.0 | **0.003**** | 664.5 | 0.520 | 1049.0 | **< 0.001***** | 569.5 | 0.165 | 1416.0 |
| DL - CS | 0.187 | 1826.5 | **< 0.001***** | 988.5 | 0.101 | 1792.5 | 0.704 | 2480.0 | 0.619 | 2552.0 |

Significant outcomes are shown in bold.

**Table S7:** Analysis of variance (ANOVA) and Tukey multiple comparisons of means of 3 song variables that followed normal distribution for natural songs of males in DL, BJ, CS and NC.

|  | **F_max_** | **F_peak_** | **RS** |
| --- | --- | --- | --- |
| *F*_3, 207_ | 22.170 | 26.180 | 3.258 |
| df | 3 | 3 | 3 |
| *P* | **< 0.001** | **< 0.001** | **0.023** |
| ***Tukey multiple comparisons of means*** | | | |
| CS - BJ | **< 0.001**  **< 0.001**  **< 0.001**  0.590  0.239  0.828 | **< 0.001**  **0.011**  0.418  **< 0.001**  **< 0.001**  0.609 | 0.131  **0.017**  0.764  0.810  0.765  0.324 |
| DL - BJ |  |  |  |
| NC - BJ |  |  |  |
| DL - CS |  |  |  |
| NC - CS |  |  |  |
| NC - DL |  |  |  |

Significant outcomes are shown in bold.

**Table S8:** Number of males sung in response to speaker and took into analyses.

| **Populations** | **Stimuli** | **Number of males sung in response to speaker** | **Number of males took into analyses** |
| --- | --- | --- | --- |
| **Colonial population** | | | |
| Dalian (DL) | Dalian (DL) | 18 | 14 |
|  | Jinan (JN) | 10 | 8 |
|  | Zhengzhou (ZZ) | 12 | 11 |
|  | Changsha (CS) | 5 | 0 |
|  | **In total** | **19** | **14** |
| Beijing (BJ) | Beijing (BJ) | 10 | 5 |
|  | Beidaihe (BDH) | 15 | 8 |
|  | Xian (XA) | 5 | 4 |
|  | Nanchong (NC) | 1 | 0 |
|  | **In total** | **16** | **11** |
| **Source population** | | | |
| Changsha (CS) | Changsha (CS) | 15 | 12 |
|  | Wuhan (WH) | 0 | 0 |
|  | Yaan (YA) | 0 | 0 |
|  | Dalian (DL) | 8 | 6 |
|  | **In total** | **18** | **15** |
| Nanchong (NC) | Nanchong (NC) | 14 | 8 |
|  | Yaan (YA) | 0 | 0 |
|  | Wuhan (WH) | 1 | 0 |
|  | Beijing (BJ) | 0 | 0 |
|  | **In total** | **14** | **8** |

**Table S9:** Eight natural and responsive song variables to each stimulus for DL, BJ, CS and NC during three periods. Number in parentheses indicates the number of males sung in response to stimuli we analyzed. Means are shown ± SD.

| **Population** | **Period** | **D (s)** | **F_max_ (kHz)** | **F_min_ (kHz)** | **F_b_ (kHz)** | **F_peak_ (kHz)** | **NS** | **RS (number/s)** | **NUS** |
| --- | --- | --- | --- | --- | --- | --- | --- | --- | --- |
| DL | **DL stimulus** | | | | | | | | |
|  | Pre-playback | 1.237 ± 0.306  (*N* = 14) | 3.614 ± 0.221  (*N* = 14) | 1.469 ± 0.145  (*N* = 14) | 2.145 ± 0.243  (*N* = 14) | 2.979 ± 0.235  (*N* = 14) | 4.791 ± 1.388  (*N* = 14) | 3.867 ± 0.635  (*N* = 14) | 3.985 ± 1.376  (*N* = 14) |
|  | During-playback | 1.145 ± 0.340  (*N* = 8) | 3.722 ± 0.328  (*N* = 8) | 1.461 ± 0.144  (*N* = 8) | 2.291 ± 0.280  (*N* = 8) | 3.044 ± 0.280  (*N* = 8) | 4.212 ± 1.244  (*N* = 8) | 3.691 ± 0.475  (*N* = 8) | 3.545 ± 1.348  (*N* = 8) |
|  | Post-playback | 1.148 ± 0.260  (*N* = 9) | 3.620 ± 0.217  (*N* = 9) | 1.457 ± 0.178  (*N* = 9) | 2.164 ± 0.294  (*N* = 9) | 2.969 ± 0.240  (*N* = 9) | 4.789 ± 1.379  (*N* = 9) | 4.145 ± 0.552  (*N* = 9) | 4.000 ± 1.433  (*N* = 9) |
|  | **JN stimulus** | | | | | | | | |
|  | Pre-playback | 0.996 ± 0.327  (*N* = 8) | 3.401 ± 0.200  (*N* = 8) | 1.523 ± 0.126  (*N* = 8) | 1.878 ± 0.238  (*N* = 8) | 2.955 ± 0.170  (*N* = 8) | 3.806 ± 0.873  (*N* = 8) | 3.926 ± 0.691  (*N* = 8) | 3.484 ± 0.926  (*N* = 8) |
|  | During-playback | 0.820 ± 0.339  (*N* = 6) | 3.372 ± 0.431  (*N* = 6) | 1.385 ± 0.103  (*N* = 6) | 1.987 ± 0.463  (*N* = 6) | 2.757 ± 0.326  (*N* = 6) | 3.542 ± 1.103  (*N* = 6) | 4.458 ± 0.726  (*N* = 6) | 3.333 ± 1.007  (*N* = 6) |
|  | Post-playback | 0.804 ± 0.218  (*N* = 6) | 3.394 ± 0.287  (*N* = 6) | 1.479 ± 0.275  (*N* = 6) | 1.915 ± 0.307  (*N* = 6) | 2.813 ± 0.317  (*N* = 6) | 3.500 ± 1.012  (*N* = 6) | 4.382 ± 0.643  (*N* = 6) | 3.318 ± 1.129  (*N* = 6) |
|  | **ZZ stimulus** | | | | | | | | |
|  | Pre-playback | 0.971 ± 0.281  (*N* = 11) | 3.402 ± 0.183  (*N* = 11) | 1.508 ± 0.123  (*N* = 11) | 1.894 ± 0.227  (*N* = 11) | 2.949 ± 0.184  (*N* = 11) | 3.795 ± 0.795  (*N* = 11) | 3.998 ± 0.674  (*N* = 11) | 3.477 ± 0.902  (*N* = 11) |
|  | During-playback | 0.763 ± 0.244  (*N* = 11) | 3.314 ± 0.296  (*N* = 11) | 1.418 ± 0.283  (*N* = 11) | 1.896 ± 0.303  (*N* = 11) | 2.714 ± 0.368  (*N* = 11) | 3.133 ± 0.944  (*N* = 11) | 4.320 ± 0.992  (*N* = 11) | 2.889 ± 1.071  (*N* = 11) |
|  | Post-playback | 0.999 ± 0.239  (*N* = 5) | 3.584 ± 0.300  (*N* = 5) | 1.392 ± 0.165  (*N* = 5) | 2.192 ± 0.287  (*N* = 5) | 2.846 ± 0.234  (*N* = 5) | 3.850 ± 0.875  (*N* = 5) | 3.885 ± 0.585  (*N* = 5) | 3.700 ± 1.031  (*N* = 5) |
| BJ | **BJ stimulus** | | | | | | | | |
|  | Pre-playback | 1.131 ± 0.331  (*N* = 5) | 3.808 ± 0.197  (*N* = 5) | 1.571 ± 0.172  (*N* = 5) | 2.237 ± 0.227  (*N* = 5) | 3.101 ± 0.302  (*N* = 5) | 4.000 ± 0.775  (*N* = 5) | 3.610 ± 0.524  (*N* = 5) | 3.524 ± 0.814  (*N* = 5) |
|  | During-playback | 1.007 ± 0.101  (*N* = 3) | 3.953 ± 0.153  (*N* = 3) | 1.485 ± 0.129  (*N* = 3) | 2.468 ± 0.240  (*N* = 3) | 3.207 ± 0.303  (*N* = 3) | 3.800 ± 0.414  (*N* = 3) | 3.780 ± 0.174  (*N* = 3) | 3.800 ± 0.414  (*N* = 3) |
|  | Post-playback | 1.018 ± 0.033  (*N* = 3) | 3.867 ± 0.149  (*N* = 3) | 1.665 ± 0.122  (*N* = 3) | 2.202 ± 0.167  (*N* = 3) | 3.077 ± 0.288  (*N* = 3) | 4.000  (*N* = 3) | 3.933 ± 0.135  (*N* = 3) | 4.000  (*N* = 3) |
|  | **BDH stimulus** | | | | | | | | |
|  | Pre-playback | 1.155 ± 0.285  (*N* = 8) | 3.912 ± 0.142  (*N* = 8) | 1.568 ± 0.178  (*N* = 8) | 2.345 ± 0.197  (*N* = 8) | 3.150 ± 0.284  (*N* = 8) | 4.065 ± 0.680  (*N* = 8) | 3.574 ± 0.446  (*N* = 8) | 3.677 ± 0.832  (*N* = 8) |
|  | During-playback | 1.101 ± 0.377  (*N* = 7) | 3.826 ± 0.249  (*N* = 7) | 1.530 ± 0.154  (*N* = 7) | 2.295 ± 0.316  (*N* = 7) | 3.084 ± 0.345  (*N* = 7) | 3.846 ± 1.120  (*N* = 7) | 3.596 ± 0.662  (*N* = 7) | 3.308 ± 0.970  (*N* = 7) |
|  | Post-playback | 1.149 ± 0.309  (*N* = 4) | 3.908 ± 0.168  (*N* = 4) | 1.535 ± 0.221  (*N* = 4) | 2.373 ± 0.282  (*N* = 4) | 3.029 ± 0.394  (*N* = 4) | 3.947 ± 1.177  (*N* = 4) | 3.421 ± 0.430  (*N* = 4) | 3.263 ± 1.195  (*N* = 4) |
|  | **XA stimulus** |  |  |  |  |  |  |  |  |
|  | Pre-playback | 1.131 ± 0.171  (*N* = 4) | 3.931 ± 0.116  (*N* = 4) | 1.634 ± 0.158  (*N* = 4) | 2.297 ± 0.162  (*N* = 4) | 3.066 ± 0.310  (*N* = 4) | 4.143 ± 0.363  (*N* = 4) | 3.693 ± 0.358  (*N* = 4) | 4.000 ± 0.679  (*N* = 4) |
|  | During-playback | 0.922 ± 0.165  (*N* = 4) | 3.963 ± 0.098  (*N* = 4) | 1.689 ± 0.155  (*N* = 4) | 2.274 ± 0.221  (*N* = 4) | 3.064 ± 0.344  (*N* = 4) | 3.750 ± 0.577  (*N* = 4) | 4.088 ± 0.403  (*N* = 4) | 3.750 ± 0.577  (*N* = 4) |
| CS | **CS stimulus** |  |  |  |  |  |  |  |  |
|  | Pre-playback | 1.288 ± 0.287  (*N* = 12) | 3.513 ± 0.211  (*N* = 12) | 1.329 ± 0.092  (*N* = 12) | 2.184 ± 0.240  (*N* = 12) | 2.680 ± 0.229  (*N* = 12) | 4.887 ± 1.068  (*N* = 12) | 3.821 ± 0.415  (*N* = 12) | 4.189 ± 0.982  (*N* = 12) |
|  | During-playback | 1.246 ± 0.255  (*N* = 9) | 3.613 ± 0.247  (*N* = 9) | 1.290 ± 0.094  (*N* = 9) | 2.323 ± 0.281  (*N* = 9) | 2.624 ± 0.249  (*N* = 9) | 4.588 ± 1.019  (*N* = 9) | 3.679 ± 0.317  (*N* = 9) | 3.912 ± 0.933  (*N* = 9) |
|  | Post-playback | 1.244 ± 0.270  (*N* = 10) | 3.618 ± 0.235  (*N* = 10) | 1.306 ± 0.083  (*N* = 10) | 2.313 ± 0.275  (*N* = 10) | 2.612 ± 0.258  (*N* = 10) | 4.786 ± 1.048  (*N* = 10) | 3.852 ± 0.304  (*N* = 10) | 3.833 ± 0.794  (*N* = 10) |
|  | **DL stimulus** |  |  |  |  |  |  |  |  |
|  | Pre-playback | 1.279 ± 0.256  (*N* = 6) | 3.617 ± 0.245  (*N* = 6) | 1.315 ± 0.081  (*N* = 6) | 2.303 ± 0.212  (*N* = 6) | 2.744 ± 0.295  (*N* = 6) | 4.607 ± 0.832  (*N* = 6) | 3.625 ± 0.281  (*N* = 6) | 4.250 ± 0.799  (*N* = 6) |
|  | During-playback | 1.196 ± 0.322  (*N* = 5) | 3.635 ± 0.213  (*N* = 5) | 1.312 ± 0.110  (*N* = 5) | 2.323 ± 0.190  (*N* = 5) | 2.652 ± 0.237  (*N* = 5) | 4.190 ± 1.167  (*N* = 5) | 3.514 ± 0.263  (*N* = 5) | 3.381 ± 0.973  (*N* = 5) |
|  | Post-playback | 1.161 ± 0.264  (*N* = 3) | 3.547 ± 0.158  (*N* = 3) | 1.388 ± 0.104  (*N* = 3) | 2.159 ± 0.132  (*N* = 3) | 2.717 ± 0.287  (*N* = 3) | 4.077 ± 0.862  (*N* = 3) | 3.538 ± 0.333  (*N* = 3) | 3.538 ± 1.127  (*N* = 3) |
| NC | **NC stimulus** |  |  |  |  |  |  |  |  |
|  | Pre-playback | 1.113 ± 0.178  (*N* = 8) | 3.644 ± 0.187  (*N* = 8) | 1.440 ± 0.158  (*N* = 8) | 2.205 ± 0.217  (*N* = 8) | 3.044 ± 0.254  (*N* = 8) | 4.088 ± 0.900  (*N* = 8) | 3.679 ± 0.423  (*N* = 8) | 3.912 ± 0.514  (*N* = 8) |
|  | During-playback | 1.054 ± 0.240  (*N* = 8) | 3.641 ± 0.150  (*N* = 8) | 1.397 ± 0.123  (*N* = 8) | 2.244 ± 0.143  (*N* = 8) | 2.983 ± 0.338  (*N* = 8) | 3.900 ± 0.712  (*N* = 8) | 3.750 ± 0.369  (*N* = 8) | 3.867 ± 0.629  (*N* = 8) |
|  | Post-playback | 1.072 ± 0.099  (*N* = 3) | 3.785 ± 0.149  (*N* = 3) | 1.485 ± 0.159  (*N* = 3) | 2.300 ± 0.239  (*N* = 3) | 2.906 ± 0.355  (*N* = 3) | 4.000  (*N* = 3) | 3.762 ± 0.352  (*N* = 3) | 4.000  (*N* = 3) |

**Table S10:** Kruskal-Wallis rank sum test of eight natural and responsive song variables to stimuli in playback experiments from both colonial and source populations during three periods.

| **Population** |  | **D** | **F_max_** | **F_min_** | **F_b_** | **F_peak_** | **NS** | **RS** | **NUS** |
| --- | --- | --- | --- | --- | --- | --- | --- | --- | --- |
| DL | **DL stimulus** | | | | | | | | |
|  | *H* | 3.740 | 7.800 | 0.046 | 7.215 | 6.403 | 4.614 | 10.834 | 1.873 |
|  | *df* | 2 | 2 | 2 | 2 | 2 | 2 | 2 | 2 |
|  | *P* | 0.154 | **0.020** | 0.977 | **0.027** | **0.041** | 0.100 | **0.004** | 0.392 |
|  | **JN stimulus** | | | | | | | | |
|  | *H* | 11.762 | 0.027 | 12.684 | 3.463 | 5.496 | 0.233 | 6.465 | 0.177 |
|  | *df* | 2 | 2 | 2 | 2 | 2 | 2 | 2 | 2 |
|  | *P* | **0.003** | 0.987 | **0.002** | 0.177 | 0.064 | 0.890 | **0.039** | 0.916 |
|  | **ZZ stimulus** | | | | | | | | |
|  | *H* | 17.295 | 9.727 | 22.573 | 15.85 | 11.766 | 11.766 | 5.168 | 9.676 |
|  | *df* | 2 | 2 | 2 | 2 | 2 | 2 | 2 | 2 |
|  | *P* | **< 0.001** | **0.008** | **< 0.001** | **< 0.001** | **0.003** | **0.003** | 0.075 | **0.008** |
| BJ | **BJ stimulus** | | | | | | | | |
|  | *H* | 0.953 | 4.351 | 10.066 | 9.534 | 2.723 | 1.858 | 4.684 | 5.165 |
|  | *df* | 2 | 2 | 2 | 2 | 2 | 2 | 2 | 2 |
|  | *P* | 0.621 | 0.114 | **0.007** | **0.009** | 0.256 | 0.395 | 0.096 | 0.076 |
|  | **BDH stimulus** | | | | | | | | |
|  | *H* | 1.412 | 1.896 | 0.843 | 0.130 | 0.849 | 0.985 | 1.804 | 2.575 |
|  | *df* | 2 | 2 | 2 | 2 | 2 | 2 | 2 | 2 |
|  | *P* | 0.494 | 0.388 | 0.656 | 0.937 | 0.654 | 0.611 | 0.406 | 0.276 |
|  | **XA stimulus** | | | | | | | | |
|  | *H* | 8.517 | 0.544 | 2.054 | 0.987 | 0.174 | 5.574 | 6.747 | 2.922 |
|  | *df* | 1 | 1 | 1 | 1 | 1 | 1 | 1 | 1 |
|  | *P* | **0.004** | 0.461 | 0.152 | 0.320 | 0.677 | **0.018** | **0.009** | 0.087 |
| CS | **CS stimulus** | | | | | | | | |
|  | *H* | 1.269 | 6.104 | 4.958 | 6.225 | 1.622 | 1.297 | 5.559 | 3.359 |
|  | *df* | 2 | 2 | 2 | 2 | 2 | 2 | 2 | 2 |
|  | *P* | 0.530 | **0.047** | 0.084 | **0.044** | 0.445 | 0.523 | 0.062 | 0.187 |
|  | **DL stimulus** | | | | | | | | |
|  | *H* | 2.892 | 1.756 | 4.635 | 7.860 | 1.460 | 4.973 | 2.430 | 9.762 |
|  | *df* | 2 | 2 | 2 | 2 | 2 | 2 | 2 | 2 |
|  | *P* | 0.236 | 0.416 | 0.099 | **0.020** | 0.482 | 0.083 | 0.297 | **0.008** |
| NC | **NC stimulus** | | | | | | | | |
|  | *H* | 1.926 | 7.174 | 2.714 | 1.301 | 2.143 | 0.538 | 1.202 | 0.538 |
|  | df | 2 | 2 | 2 | 2 | 2 | 2 | 2 | 2 |
|  | *P* | 0.382 | **0.028** | 0.257 | 0.522 | 0.342 | 0.764 | 0.548 | 0.764 |

Significant outcomes are shown in bold.

**Table S11:** Multiple comparisons (Wilcoxon rank sum tests) of natural and responsive song variables to variant stimuli under playback experiments measured from both colonial and source populations during three periods. Probability adjustment = “ holm ”.

| **Population** | **Stimuli** | **Variables** | **Pre - During** | ***T*** | **During - Post** | ***T*** | **Pre - Post** | ***T*** |
| --- | --- | --- | --- | --- | --- | --- | --- | --- |
| DL | DL | F_max_ | **0.023** | 753.0 | 0.056 | 436.0 | 0.719 | 1237.0 |
|  |  | F_b_ | **0.022** | 750.5 | 0.108 | 459.5 | 0.823 | 1257.5 |
|  |  | F_peak_ | **0.046** | 786.5 | 0.088 | 452.0 | 0.963 | 1284.5 |
|  |  | RS | 0.150 | 923.0 | **0.002** | 337.0 | 0.062 | 965.0 |
|  | JN | D | **0.007** | 191.0 | 0.733 | 280.0 | **0.016** | 193.5 |
|  |  | F_min_ | **< 0.001** | 135.0 | 0.398 | 223.5 | 0.398 | 269.5 |
|  |  | RS | 0.065 | 236.5 | 0.595 | 288.5 | 0.120 | 236.5 |
|  | ZZ | D | **< 0.001** | 537.5 | **0.004** | 229.5 | 0.643 | 407.5 |
|  |  | F_max_ | 0.105 | 792.0 | **0.011** | 245.5 | 0.078 | 297.0 |
|  |  | F_min_ | **< 0.001** | 443.0 | 0.564 | 409.0 | **0.003** | 223.0 |
|  |  | F_b_ | 0.774 | 954.5 | **0.001** | 209.0 | **< 0.001** | 174.5 |
|  |  | F_peak_ | **0.003** | 584.5 | 0.238 | 366.5 | 0.151 | 317.0 |
|  |  | NS | **0.008** | 655.0 | **0.017** | 278.0 | 0.665 | 467.5 |
|  |  | NUS | **0.031** | 697.5 | **0.031** | 280.5 | 0.486 | 484.5 |
| BJ | BJ | F_min_ | 0.176 | 111.5 | **0.003** | 33.5 | 0.176 | 104.0 |
|  |  | F_b_ | **0.021** | 75.5 | **0.021** | 47.0 | 0.596 | 140.5 |
| CS | CS | F_max_ | 0.162 | 700.0 | 0.059 | 698.0 | 0.871 | 801.5 |
|  |  | F_b_ | 0.090 | 670.0 | 0.958 | 708.5 | 0.090 | 823.0 |
|  | DL | F_b_ | 0.693 | 274.0 | **0.016** | 57.5 | **0.049** | 101.5 |
|  |  | NUS | **0.008** | 151.5 | 0.672 | 124.5 | 0.102 | 115.5 |
| NC | NC | F_max_ | 0.925 | 517.5 | **0.035** | 105.0 | **0.035** | 114.5 |

Significant outcomes are shown in bold.

**Table S12:** Kruskal-Wallis rank sum test of number of responsive songs (NOS), mean song length (LM), and total song length (LT) among different stimuli of during- and post-playback periods in DL, BJ and CS populations. We didn’t compare these variables for WH and YA stimuli because no CS males sang in response to them.

|  | **Period** | **NOS** | **LM** | **LT** |  | **NOS** | **LM** | **LT** |  | **NOS** | **LM** | **LT** |
| --- | --- | --- | --- | --- | --- | --- | --- | --- | --- | --- | --- | --- |
| **DL** | **During-playback** | | | | **BJ** | | | | **CS** | | | |
|  | *H* | 12.146 | 10.531 | 11.866 |  | 16.154 | 17.374 | 16.057 |  | 5.290 | 5.519 | 5.566 |
|  | *df* | 3 | 3 | 3 |  | 3 | 3 | 3 |  | 1 | 1 | 1 |
|  | *P*  **Post-playback** | **0.007** | **0.015** | **0.008** |  | **0.001** | **< 0.001** | **0.001** |  | **0.021** | **0.019** | **0.018** |
|  | *H* | 15.086 | 16.945 | 15.765 |  | 16.553 | 17.404 | 16.733 |  | 3.729 | 1.975 | 3.670 |
|  | *df* | 3 | 3 | 3 |  | 3 | 3 | 3 |  | 1 | 1 | 1 |
|  | *P* | **0.002** | **< 0.001** | **< 0.001** |  | **< 0.001** | **< 0.001** | **< 0.001** |  | 0.053 | 0.160 | 0.055 |

Significant outcomes are shown in bold.

**Table S13:** Multiple comparisons (Wilcoxon rank sum tests) of number of responsive songs (NOS), mean song length (LM), and total song length (LT) among different stimuli of during- and post-playback periods in DL and BJ populations.

| **Variables** |  | **DL** | | | |  | **BJ** | | | |
| --- | --- | --- | --- | --- | --- | --- | --- | --- | --- | --- |
|  |  | **During-playback** | | **Post-playback** | |  | **During-playback** | | **Post-playback** | |
|  | **Stimuli** | ***P*** | ***T*** | ***P*** | ***T*** | **Stimuli** | ***P*** | ***T*** | ***P*** | ***T*** |
| **NOS** | CS - JN | 0.239 | 139.0 | **0.023** | 130.0 | BJ - NC | **0.020** | 281.0 | 0.060 | 250.0 |
|  | CS - ZZ | **0.021** | 103.0 | **0.038** | 140.0 | BJ - XA | 0.403 | 232.0 | 0.060 | 250.0 |
|  | CS - DL | **0.009** | 90.5 | **< 0.001** | 90.0 | BJ - BDH | 0.403 | 154.5 | 0.416 | 225.5 |
|  | JN - ZZ | 0.735 | 167.5 | 0.897 | 204.5 | NC - XA | 0.212 | 158.0 | NA | 200.0 |
|  | JN - DL | 0.414 | 146.5 | 0.290 | 143.5 | NC - BDH | **< 0.001** | 76.0 | **0.010** | 280.0 |
|  | ZZ - DL | 0.735 | 184.5 | 0.290 | 145.0 | XA - BDH | 0.158 | 130.0 | **0.010** | 280.0 |
| **LM** | CS - JN | 0.322 | 154.5 | **0.023** | 130.0 | BJ - NC | **0.022** | 280.5 | 0.059 | 250.0 |
|  | CS - ZZ | 0.217 | 131.5 | **0.038** | 140.0 | BJ - XA | 0.257 | 238.0 | 0.059 | 250.0 |
|  | CS - DL | **0.020** | 99.5 | **< 0.001** | 90.0 | BJ - BDH | 0.257 | 146.0 | 0.193 | 240.5 |
|  | JN - ZZ | 0.562 | 179.0 | 0.871 | 205.5 | NC - XA | 0.244 | 159.5 | NA | 200.0 |
|  | JN - DL | 0.219 | 131.0 | 0.109 | 130.5 | NC - BDH | **< 0.001** | 78.5 | **0.010** | 280.0 |
|  | ZZ - DL | 0.318 | 141.0 | 0.109 | 130.0 | XA - BDH | 0.060 | 117.5 | **0.010** | 280.0 |
| **LT** | CS - JN | 0.337 | 144.0 | **0.023** | 130.0 | BJ - NC | **0.028** | 278.5 | 0.059 | 250.0 |
|  | CS - ZZ | **0.035** | 108.5 | **0.038** | 140.0 | BJ - XA | 0.341 | 230.5 | 0.059 | 250.0 |
|  | CS - DL | **0.009** | 91.0 | **0.001** | 90.0 | BJ - BDH | 0.312 | 149.5 | 0.354 | 229.0 |
|  | JN - ZZ | 0.713 | 169.0 | 0.884 | 205.0 | NC - XA | 0.202 | 157.5 | NA | 200.0 |
|  | JN - DL | 0.352 | 143.5 | 0.207 | 138.5 | NC - BDH | **< 0.001** | 76.5 | **0.010** | 280.0 |
|  | ZZ - DL | 0.713 | 166.0 | 0.207 | 139.0 | XA - BDH | 0.164 | 130.5 | **0.010** | 280.0 |

Significant outcomes are shown in bold.

**Table** **S14:** Loadings and cumulative proportion for principal components on behavioral response variables in the analysis of playback experiments in during-playback period.

| **Response variable** | **DL** | |  | **BJ** | |  | **CS** | |  | **NC** | |
| --- | --- | --- | --- | --- | --- | --- | --- | --- | --- | --- | --- |
|  | **PC1**  **(59.8%)** | **PC2**  **(21.4%)** |  | **PC1**  **(65.1%)** | **PC2**  **(18.3%)** |  | **PC1**  **(68.3%)** | **PC2**  **(21.4%)** |  | **PC1**  **(68.4%)** | **PC2**  **(18.3%)** |
| Cumulative proportion  (PC1 + PC2) | 81.2% | |  | 83.4% | |  | 89.7% | |  | 86.7% | |
| Eigenvalue | 2.319 | 1.389 |  | 2.421 | 1.285 |  | 2.478 | 1.388 |  | 2.482 | 1.283 |
| SS | **0.349** | **0.305** |  | **0.354** | 0.227 |  | **0.369** | 0.231 |  | **0.368** | 0.196 |
| LA | -**0.354** | **-**0.243 |  | -**0.363** | **-**0.234 |  | -**0.374** | -0.228 |  | -**0.323** | -**0.324** |
| DR | **0.391** | 0.204 |  | **0.371** | 0.235 |  | **0.372** | 0.239 |  | **0.378** | 0.164 |
| AD | -**0.322** | -**0.313** |  | -**0.326** | -**0.351** |  | -**0.343** | **-**0.260 |  | -**0.319** | -**0.366** |
| NOC | 0.294 | **0.341** |  | 0.247 | **0.414** |  | 0.213 | **0.512** |  | 0.168 | **0.529** |
| LS | -**0.336** | 0.296 |  | -**0.341** | 0.283 |  | -**0.334** | **0.312** |  | -**0.357** | **0.306** |
| NOS | **0.317** | -**0.430** |  | **0.321** | -**0.426** |  | **0.319** | -**0.389** |  | **0.343** | -**0.353** |
| LM | **0.342** | -**0.301** |  | **0.350** | **-**0.269 |  | **0.337** | -**0.313** |  | **0.361** | **-**0.253 |
| LT | 0.282 | -**0.478** |  | **0.310** | -**0.459** |  | **0.309** | -**0.401** |  | **0.335** | -**0.366** |

PC1 and PC2 are the first and second principal components of response variables in playback experiments.

**Table S15:** Results of linear mixed models showing the effect of different stimuli on behavioral response intensity in during-playback period for each population.

| **Populations** | **Stimuli** | **Estimate** | **SE** | ***df*** | ***t*** | ***P*** |
| --- | --- | --- | --- | --- | --- | --- |
| **Source populations** | | | | | | |
| CS | (Intercept) | -1.518 | 0.317 | 100.000 | -4.794 | **< 0.001** |
|  | CS | 5.335 | 0.448 | 100.000 | 11.911 | **< 0.001** |
|  | WH | 0.178 | 0.448 | 100.000 | 0.397 | 0.692 |
|  | YA | 0.156 | 0.448 | 100.000 | 0.349 | 0.728 |
|  | DL | 1.923 | 0.448 | 100.000 | 4.292 | **< 0.001** |
| NC | (Intercept) | -1.197 | 0.270 | 99.850 | -4.428 | **< 0.001** |
|  | NC | 5.528 | 0.379 | 80.000 | 14.604 | **< 0.001** |
|  | YA | 0.088 | 0.379 | 80.000 | 0.232 | 0.817 |
|  | WH | 0.273 | 0.379 | 80.000 | 0.720 | 0.473 |
|  | BJ | 0.096 | 0.379 | 80.000 | 0.253 | 0.801 |
| **Colonial populations** | | | | | | |
| DL | (Intercept) | -2.623 | 0.340 | 92.089 | -7.718 | **< 0.001** |
|  | DL | 4.854 | 0.444 | 80.000 | 10.933 | **< 0.001** |
|  | JN | 3.236 | 0.444 | 80.000 | 7.289 | **< 0.001** |
|  | ZZ | 3.754 | 0.444 | 80.000 | 8.456 | **< 0.001** |
|  | CS | 1.269 | 0.444 | 80.000 | 2.857 | **0.005** |
| BJ | (Intercept) | -2.060 | 0.383 | 94.912 | -5.378 | **< 0.001** |
|  | BJ | 3.987 | 0.509 | 80.000 | 7.825 | **< 0.001** |
|  | BDH | 4.084 | 0.509 | 80.000 | 8.017 | **< 0.001** |
|  | XA | 1.749 | 0.509 | 80.000 | 3.433 | **< 0.001** |
|  | NC | 0.482 | 0.509 | 80.000 | 0.945 | 0.347 |

Significant outcomes are shown in bold.

**Table S16:** Results of Tukey's test post hoc comparison of behavioral response by source and colonial populations of light-vented bulbul in during-playback period.

| **Populations** | **Pairwise comparison** | **Estimate** | **SE** | ***df*** | ***t*** | ***P*** |
| --- | --- | --- | --- | --- | --- | --- |
| **Source populations** | | | | | | |
| CS | CS - WH | 5.157 | 0.460 | 84.2 | 11.222 | **< 0.001** |
|  | CS - YA | -5.179 | 0.460 | 84.2 | -11.269 | **< 0.001** |
|  | CS - DL | 3.413 | 0.460 | 84.2 | 7.425 | **< 0.001** |
|  | CS - GT | -5.335 | 0.460 | 84.2 | -11.609 | **< 0.001** |
|  | WH - YA | -0.021 | 0.460 | 84.2 | -0.046 | 1.000 |
|  | WH - DL | -1.745 | 0.460 | 84.2 | -3.797 | **0.003** |
|  | WH - GT | -0.178 | 0.460 | 84.2 | -0.387 | 0.995 |
|  | YA - DL | -1.766 | 0.460 | 84.2 | -3.843 | **0.002** |
|  | YA - GT | -0.156 | 0.460 | 84.2 | -0.340 | 0.997 |
|  | DL - GT | -1.923 | 0.460 | 84.2 | -4.184 | **< 0.001** |
| NC | NC - YA | 5.440 | 0.388 | 84.2 | 14.009 | **< 0.001** |
|  | NC - WH | -5.255 | 0.388 | 84.2 | -13.533 | **< 0.001** |
|  | NC - BJ | 5.432 | 0.388 | 84.2 | 13.988 | **< 0.001** |
|  | NC - GT | -5.528 | 0.388 | 84.2 | -14.235 | **< 0.001** |
|  | YA - WH | 0.185 | 0.388 | 84.2 | 0.476 | 0.989 |
|  | YA - BJ | -0.008 | 0.388 | 84.2 | -0.021 | 1.000 |
|  | YA - GT | -0.088 | 0.388 | 84.2 | -0.226 | 0.999 |
|  | WH - BJ | 0.177 | 0.388 | 84.2 | 0.455 | 0.991 |
|  | WH - GT | -0.273 | 0.388 | 84.2 | -0.702 | 0.956 |
|  | BJ - GT | -0.096 | 0.388 | 84.2 | -0.247 | 0.999 |
| **Colonial populations** | | | | | | |
| DL | DL - ZZ | -1.100 | 0.456 | 84.2 | -2.415 | 0.121 |
|  | DL - JN | 1.618 | 0.456 | 84.2 | 3.552 | **0.006** |
|  | DL - CS | 3.586 | 0.456 | 84.2 | 7.872 | **< 0.001** |
|  | DL - GT | -4.854 | 0.456 | 84.2 | -10.657 | **< 0.001** |
|  | ZZ - JN | 0.518 | 0.456 | 84.2 | 1.137 | 0.787 |
|  | ZZ - CS | 2.486 | 0.456 | 84.2 | 5.457 | **< 0.001** |
|  | ZZ - GT | -3.754 | 0.456 | 84.2 | -8.242 | **< 0.001** |
|  | JN - CS | 1.968 | 0.456 | 84.2 | 4.320 | **< 0.001** |
|  | JN - GT | -3.236 | 0.456 | 84.2 | -7.105 | **< 0.001** |
|  | CS - GT | -1.269 | 0.456 | 84.2 | -2.785 | 0.050 |
| BJ | BJ - BDH | -0.098 | 0.523 | 84.2 | -0.187 | 0.999 |
|  | BJ - XA | -2.238 | 0.523 | 84.2 | -4.281 | **< 0.001** |
|  | BJ - NC | 3.505 | 0.523 | 84.2 | 6.706 | **< 0.001** |
|  | BJ - GT | -3.987 | 0.523 | 84.2 | -7.627 | **< 0.001** |
|  | BDH - XA | -2.335 | 0.523 | 84.2 | -4.468 | **< 0.001** |
|  | BDH - NC | 3.603 | 0.523 | 84.2 | 6.893 | **< 0.001** |
|  | BDH - GT | -4.084 | 0.523 | 84.2 | -7.814 | **< 0.001** |
|  | XA - NC | 1.267 | 0.523 | 84.2 | 2.425 | 0.119 |
|  | XA - GT | -1.749 | 0.523 | 84.2 | -3.346 | **0.011** |
|  | NC - GT | -0.482 | 0.523 | 84.2 | -0.921 | 0.888 |

Significant outcomes are shown in bold.

**Table S17:** Loadings and cumulative proportion for principal components on behavioral response variables in the analysis of playback experiments in post-playback period.

| **Response variable** | **DL** | |  | **BJ** | |  | **CS** | |  | **NC** | |
| --- | --- | --- | --- | --- | --- | --- | --- | --- | --- | --- | --- |
|  | **PC1**  **(62.4%)** | **PC2**  **(14.8%)** |  | **PC1**  **(55.9%)** | **PC2**  **(18.5%)** |  | **PC1**  **(72.3%)** | **PC2**  **(15.2%)** |  | **PC1**  **(65.9%)** | **PC2**  **(18.5%)** |
| Cumulative proportion  (PC1 + PC2) | 77.2% | |  | 74.4% | |  | 87.5% | |  | 84.4% | |
| Eigenvalue | 2.369 | 1.153 |  | 2.243 | 1.291 |  | 2.551 | 1.171 |  | 2.435 | 1.291 |
| SS | **0.350** | 0.230 |  | **0.379** | 0.137 |  | **0.347** | 0.231 |  | **0.341** | 0.171 |
| LA | -**0.325** | **-**0.258 |  | -**0.345** | **-**0.272 |  | -**0.348** | **-**0.193 |  | -**0.360** | **-**0.233 |
| DR | **0.358** | 0.228 |  | **0.381** | 0.212 |  | **0.366** | 0.176 |  | **0.391** | NA |
| AD | -**0.332** | **-**0.295 |  | -**0.309** | -**0.438** |  | -**0.343** | -**0.309** |  | **-**0.296 | -**0.434** |
| NOC | 0.192 | **0.537** |  | 0.215 | **0.404** |  | 0.213 | **0.587** |  | 0.198 | **0.522** |
| LS | -**0.347** | 0.135 |  | -**0.302** | 0.119 |  | -**0.339** | **0.338** |  | -**0.366** | 0.186 |
| NOS | **0.359** | -**0.372** |  | **0.349** | -**0.422** |  | **0.337** | -**0.365** |  | **0.323** | -**0.433** |
| LM | **0.361** | -**0.325** |  | **0.359** | -**0.313** |  | **0.350** | **-**0.228 |  | **0.365** | **-**0.173 |
| LT | **0.341** | -**0.438** |  | **0.329** | -**0.466** |  | **0.334** | -**0.374** |  | **0.321** | -**0.440** |

PC1 and PC2 are the first and second principal components of response variables in playback experiments.

**Table S18:** Results of linear mixed models showing the effect of different stimuli on behavioral response intensity in post-playback period for each population.

| **Populations** | **Stimuli** | **Estimate** | **SE** | ***df*** | ***t*** | ***P*** |
| --- | --- | --- | --- | --- | --- | --- |
| **Source populations** | | | | | | |
| CS | (Intercept) | -1.444 | 0.354 | 100.000 | -4.078 | **< 0.001** |
|  | CS | 5.168 | 0.501 | 100.000 | 10.321 | **< 0.001** |
|  | WH | 0.107 | 0.501 | 100.000 | 0.213 | 0.832 |
|  | YA | 0.023 | 0.501 | 100.000 | 0.045 | 0.964 |
|  | DL | 1.922 | 0.501 | 100.000 | 3.839 | **< 0.001** |
| NC | (Intercept) | -0.914 | 0.343 | 99.973 | -2.664 | **0.009** |
|  | NC | 4.695 | 0.484 | 79.999 | 9.710 | **< 0.001** |
|  | YA | -0.056 | 0.484 | 79.999 | -0.116 | 0.908 |
|  | WH | -0.056 | 0.484 | 79.999 | -0.116 | 0.907 |
|  | BJ | -0.010 | 0.484 | 79.999 | -0.020 | 0.983 |
| **Colonial populations** | | | | | | |
| DL | (Intercept) | -1.522 | 0.417 | 94.971 | -3.653 | **< 0.001** |
|  | DL | 3.748 | 0.555 | 80.000 | 6.760 | **< 0.001** |
|  | JN | 2.134 | 0.555 | 80.000 | 3.848 | **< 0.001** |
|  | ZZ | 1.912 | 0.555 | 80.000 | 3.449 | **< 0.001** |
|  | GT | -0.182 | 0.555 | 80.000 | -0.329 | 0.743 |
| BJ | (Intercept) | -1.288 | 0.385 | 94.032 | -3.344 | **0.001** |
|  | BJ | 3.100 | 0.509 | 80.000 | 6.087 | **< 0.001** |
|  | BDH | 2.987 | 0.509 | 80.000 | 5.864 | **< 0.001** |
|  | XA | 0.283 | 0.509 | 80.000 | 0.555 | 0.580 |
|  | NC | 0.071 | 0.509 | 80.000 | 0.140 | 0.889 |

Significant outcomes are shown in bold.

**Table S19:** Results of Tukey's test post hoc comparison of behavioral response by source and colonial populations of light-vented bulbul in post-playback.

| **Populations** | **Pairwise comparison** | **Estimate** | **SE** | ***df*** | ***t*** | ***P*** |
| --- | --- | --- | --- | --- | --- | --- |
| **Source populations** | | | | | | |
| CS | CS - WH | 5.061 | 0.514 | 84.2 | 9.852 | **< 0.001** |
|  | CS - YA | -5.145 | 0.514 | 84.2 | -10.015 | **< 0.001** |
|  | CS - DL | 3.246 | 0.514 | 84.2 | 6.317 | **< 0.001** |
|  | CS - GT | -5.168 | 0.514 | 84.2 | -10.059 | **< 0.001** |
|  | WH - YA | -0.084 | 0.514 | 84.2 | -0.163 | 1.000 |
|  | WH - DL | -1.816 | 0.514 | 84.2 | -3.535 | **0.006** |
|  | WH - GT | -0.107 | 0.514 | 84.2 | -0.207 | 0.999 |
|  | YA - DL | -1.900 | 0.514 | 84.2 | -3.698 | **0.004** |
|  | YA - GT | -0.023 | 0.514 | 84.2 | -0.044 | 1.000 |
|  | DL -GT | -1.922 | 0.514 | 84.2 | -3.742 | **0.003** |
| NC | NC - YA | 4.751 | 0.496 | 84.2 | 9.577 | **< 0.001** |
|  | NC - WH | -4.751 | 0.496 | 84.2 | -9.577 | **< 0.001** |
|  | NC - BJ | 4.705 | 0.496 | 84.2 | 9.484 | **< 0.001** |
|  | NC - GT | -4.695 | 0.496 | 84.2 | -9.464 | **< 0.001** |
|  | YA - WH | 0.000 | 0.496 | 84.2 | 0.000 | 1.000 |
|  | YA - BJ | -0.046 | 0.496 | 84.2 | -0.094 | 1.000 |
|  | YA - GT | 0.056 | 0.496 | 84.2 | 0.113 | 1.000 |
|  | WH - BJ | -0.046 | 0.496 | 84.2 | -0.094 | 1.000 |
|  | WH - GT | 0.056 | 0.496 | 84.2 | 0.113 | 1.000 |
|  | BJ - GT | 0.010 | 0.496 | 84.2 | 0.020 | 1.000 |
| **Colonial populations** | | | | | | |
| DL | DL - ZZ | 1.836 | 0.569 | 84.2 | 3.227 | **0.015** |
|  | DL - JN | 1.614 | 0.569 | 84.2 | 2.837 | **0.044** |
|  | DL - CS | -3.748 | 0.569 | 84.2 | -6.588 | **< 0.001** |
|  | DL - GT | 3.930 | 0.569 | 84.2 | 6.909 | **< 0.001** |
|  | ZZ - JN | 0.222 | 0.569 | 84.2 | 0.389 | 0.995 |
|  | ZZ - CS | -1.912 | 0.569 | 84.2 | -3.362 | **0.010** |
|  | ZZ - GT | -2.094 | 0.569 | 84.2 | -3.682 | **0.004** |
|  | JN - CS | -2.134 | 0.569 | 84.2 | -3.751 | **0.003** |
|  | JN - GT | -2.316 | 0.569 | 84.2 | -4.071 | **0.001** |
|  | CS - GT | 0.182 | 0.569 | 84.2 | 0.320 | 0.998 |
| BJ | BJ - BDH | 0.113 | 0.522 | 84.2 | 0.217 | 0.999 |
|  | BJ - XA | -2.817 | 0.522 | 84.2 | -5.392 | **< 0.001** |
|  | BJ - NC | 3.029 | 0.522 | 84.2 | 5.797 | **< 0.001** |
|  | BJ - GT | -3.100 | 0.522 | 84.2 | -5.933 | **< 0.001** |
|  | BDH - XA | -2.704 | 0.522 | 84.2 | -5.175 | **< 0.001** |
|  | BDH - NC | 2.916 | 0.522 | 84.2 | 5.580 | **< 0.001** |
|  | BDH - GT | -2.987 | 0.522 | 84.2 | -5.716 | **< 0.001** |
|  | XA - NC | 0.212 | 0.522 | 84.2 | 0.405 | 0.994 |
|  | XA - GT | -0.283 | 0.522 | 84.2 | -0.541 | 0.983 |
|  | NC - GT | -0.071 | 0.522 | 84.2 | -0.136 | 0.999 |

Significant outcomes are shown in bold.

**Table S20:** Matrix of Principle Content of average value of 6 variables of 10 populations (Cumulative contribution rate of PC1 and PC2 is 76.83%)

| **Variables** | **Principal Content** | |
| --- | --- | --- |
|  | **PC1** | **PC2** |
| F_max_ | -0.009 | 0.946 |
| F_min_ | -0.644 | -0.054 |
| F_peak_ | 0.765 | 0.252 |
| D | 0.864 | 0.360 |
| NS | 0.948 | -0.160 |
| RS | 0.786 | -0.480 |

**Table S21:** Matrix of Principle Content of 6 variables of all individual from 10 populations (Cumulative contribution rate of PC1 and PC2 is 53.69%)

| **Variables** | **Principle Content** | |
| --- | --- | --- |
|  | **PC1** | **PC2** |
| F_max_ | -0.051 | -0.441 |
| F_min_ | -0.337 | 0.136 |
| F_peak_ | -0.097 | 0.393 |
| D | 0.876 | -0.385 |
| NS | 0.968 | 0.168 |
| RS | 0.268 | 0.881 |

**Table S22:** Linear discriminant analysis (LDA) of natural songs of males in DL, BJ, CS and NC. Proportion of trace, SVD and coefficients of linear discriminants of 8 song variables are shown.

|  | **LD1** | **LD2** | **LD3** |
| --- | --- | --- | --- |
| Proportion of trace (%) | 73.9 | 19.4 | 6.7 |
| SVD | 6.826 | 3.491 | 2.060 |
| Coefficients of linear discriminants | | | |
| D | 1.485 | -13.857 | 3.139 |
| Fmax | -0.002 | -0.001 | -0.003 |
| Fmin | -0.005 | 0.002 | -0.003 |
| Fb | -0.000 | -0.001 | -0.001 |
| Fpeak | -0.001 | 0.001 | 0.003 |
| NS | -0.186 | 4.250 | -1.514 |
| RS | 0.944 | -3.185 | 0.924 |
| NUS | 0.261 | -0.816 | 0.821 |

**Table S23:** Kruskal-Wallis rank sum test of response variables (PC1 and PC2) in colonial and source populations.

| **Population** | ***H*** | **df** | ***P*** | ***H*** | **df** | ***P*** |
| --- | --- | --- | --- | --- | --- | --- |
| **During-playback** | | | | **Post-playback** | | |
| **DL** | | | | | | |
| PC1 | 55.737 | 4 | **< 0.001** | 41.928 | 4 | **< 0.001** |
| PC2 | 11.822 | 4 | **0.019** | 5.440 | 4 | 0.245 |
| **BJ** | | | |  |  |  |
| PC1 | 53.988 | 4 | **< 0.001** | 56.241 | 4 | **< 0.001** |
| PC2 | 18.067 | 4 | **0.001** | 25.838 | 4 | **< 0.001** |
| **CS** | | | |  |  |  |
| PC1 | 62.201 | 4 | **< 0.001** | 63.121 | 4 | **< 0.001** |
| PC2 | 2.008 | 4 | 0.734 | 5.512 | 4 | 0.239 |
| **NC** | | | |  |  |  |
| PC1 | 78.826 | 4 | **< 0.001** | 60.902 | 4 | **< 0.001** |
| PC2 | 0.647 | 4 | 0.958 | 14.942 | 4 | **0.005** |

PC1 and PC2 are the first and second principal components of response variables in playback experiments. Significant outcomes are shown in bold.

**Table S24:** Multiple comparisons (Wilcoxon rank sum tests) of behavioral response variables (PC1 and PC2) under playback experiments measured from both colonial and source populations in during-playback period. Probability adjustment = “ holm ”.

|  | **DL** | |  | **BJ** | |  | **CS** | |  | **NC** | |
| --- | --- | --- | --- | --- | --- | --- | --- | --- | --- | --- | --- |
|  | ***P* value** | |  | ***P* value** | |  | ***P* value** | |  | ***P* value** | |
|  | **PC1** | **PC2** |  | **PC1** | **PC2** |  | **PC1** | **PC2** |  | **PC1** | **PC2** |
| Stimuli | | | Stimuli | | | Stimuli | | | Stimuli | | |
| GT - CS | **< 0.001** | 0.180 | GT - NC | 0.234 | 1.000 | GT - DL | 0.074 | 1.000 | GT - BJ | 1.000 | 1.000 |
| GT - JN | **< 0.001** | 0.362 | GT - XA | **0.034** | 1.000 | GT - WH | 1.000 | 1.000 | GT - WH | 1.000 | 1.000 |
| GT - ZZ | **< 0.001** | **0.030** | GT - BJ | **< 0.001** | **0.007** | GT - YA | 1.000 | 1.000 | GT - YA | 1.000 | 1.000 |
| GT - DL | **< 0.001** | 0.056 | GT - BDH | **< 0.001** | 0.651 | GT - CS | **< 0.001** | 1.000 | GT - NC | **< 0.001** | 1.000 |
| CS - JN | **0.015** | 0.755 | NC - XA | 0.372 | 1.000 | DL - WH | 0.074 | 1.000 | BJ - WH | 1.000 | 1.000 |
| CS - ZZ | **0.005** | 0.755 | NC - BJ | **< 0.001** | **0.012** | DL - YA | **0.030** | 1.000 | BJ - YA | 1.000 | 1.000 |
| CS - DL | **< 0.001** | 0.432 | NC - BDH | **< 0.001** | 1.000 | DL - CS | **< 0.001** | 1.000 | BJ - NC | **< 0.001** | 1.000 |
| JN - ZZ | 0.397 | 1.000 | XA - BJ | **0.015** | **0.012** | WH - YA | 1.000 | 1.000 | WH - YA | 1.000 | 1.000 |
| JN - DL | **0.023** | 1.000 | XA - BDH | **0.034** | 1.000 | WH - CS | **< 0.001** | 1.000 | WH - NC | **< 0.001** | 1.000 |
| ZZ - DL | 0.397 | 1.000 | BJ - BDH | 0.525 | 0.192 | YA - CS | **< 0.001** | 1.000 | YA - NC | **< 0.001** | 1.000 |

PC1 and PC2 are the first and second principal components of response variables in playback experiments. “ GT ” indicates the stimuli of Great Tit for control groups. Significant outcomes are shown in bold.

**Table S25:** Multiple comparisons (Wilcoxon rank sum tests) of behavioral response variables (PC1 and PC2) under playback experiments measured from both colonial and source populations in post-playback period. Probability adjustment = “ holm ”.

|  | **DL** | |  | **BJ** | |  | **CS** | |  | **NC** | |
| --- | --- | --- | --- | --- | --- | --- | --- | --- | --- | --- | --- |
|  | ***P* value** | |  | ***P* value** | |  | ***P* value** | |  | ***P* value** | |
|  | **PC1** | **PC2** |  | **PC1** | **PC2** |  | **PC1** | **PC2** |  | **PC1** | **PC2** |
| Stimuli | | | Stimuli | | | Stimuli | | | Stimuli | | |
| GT - CS | 0.059 | 0.117 | GT - NC | 1.000 | 1.000 | GT - DL | 0.137 | 0.480 | GT - BJ | 1.000 | 1.000 |
| GT - ZZ | **0.002** | 1.000 | GT - XA | 1.000 | 1.000 | GT - WH | 1.000 | 1.000 | GT - WH | 1.000 | 1.000 |
| GT - JN | **0.002** | 0.182 | GT - BJ | **< 0.001** | **< 0.001** | GT - YA | 1.000 | 1.000 | GT - YA | 1.000 | 1.000 |
| GT - DL | **< 0.001** | 1.000 | GT - BDH | **< 0.001** | 0.638 | GT - CS | **< 0.001** | 1.000 | GT - NC | **< 0.001** | 0.217 |
| CS - ZZ | 0.059 | 1.000 | NC - XA | 1.000 | 1.000 | DL - WH | **0.041** | 0.710 | BJ - WH | 1.000 | 1.000 |
| CS - JN | **0.046** | 1.000 | NC - BJ | **< 0.001** | **< 0.001** | DL - YA | **0.039** | 0.739 | BJ - YA | 1.000 | 1.000 |
| CS - DL | **< 0.001** | 1.000 | NC - BDH | **< 0.001** | 0.665 | DL - CS | **0.004** | 1.000 | BJ - NC | **< 0.001** | 0.217 |
| ZZ - JN | 0.782 | 1.000 | XA - BJ | **< 0.001** | **0.005** | WH - YA | 1.000 | 1.000 | WH - YA | 1.000 | 1.000 |
| ZZ - DL | 0.073 | 1.000 | XA - BDH | **< 0.001** | 1.000 | WH - CS | **< 0.001** | 1.000 | WH - NC | **< 0.001** | 0.143 |
| JN - DL | 0.108 | 1.000 | BJ - BDH | 1.000 | 0.089 | YA - CS | **< 0.001** | 1.000 | YA - NC | **< 0.001** | 0.143 |

PC1 and PC2 are the first and second principal components of response variables in playback experiments. “ GT ” indicates the stimuli of Great Tit for control groups. Significant outcomes are shown in bold.

**Table S24:** The models and its’ AIC of linear mixed models for each population in two periods. The order of stimuli presentation was dropped from our final models.

| **Periods** | **Populations** | **Model** | **Fixed effect** | **AIC** |
| --- | --- | --- | --- | --- |
| During-playback | DL | Model 1.1 | PC1 ~ order + stimuli + (1\|indID) | 379.96 |
|  |  | Model 1.2 | PC1 ~ stimuli + (1\|indID) | 378.05 |
|  | BJ | Model 2.1 | PC1 ~ order + stimuli + (1\|indID) | 404.62 |
|  |  | Model 2.2 | PC1 ~ stimuli + (1\|indID) | 403.23 |
|  | CS | Model 3.1 | PC1 ~ order + stimuli + (1\|indID) | 366.00 |
|  |  | Model 3.2 | PC1 ~ stimuli + (1\|indID) | 367.42 |
|  | NC | Model 4.1 | PC1 ~ order + stimuli + (1\|indID) | 335.58 |
|  |  | Model 4.2 | PC1 ~ stimuli + (1\|indID) | 335.62 |
| Post-playback | DL | Model 1.1 | PC1 ~ order + stimuli + (1\|indID) | 421.78 |
|  |  | Model 1.2 | PC1 ~ stimuli + (1\|indID) | 420.11 |
|  | BJ | Model 2.1 | PC1 ~ order + stimuli + (1\|indID) | 405.86 |
|  |  | Model 2.2 | PC1 ~ stimuli + (1\|indID) | 403.94 |
|  | CS | Model 3.1 | PC1 ~ order + stimuli + (1\|indID) | 388.02 |
|  |  | Model 3.2 | PC1 ~ stimuli + (1\|indID) | 389.71 |
|  | NC | Model 4.1 | PC1 ~ order + stimuli + (1\|indID) | 385.17 |
|  |  | Model 4.2 | PC1 ~ stimuli + (1\|indID) | 383.52 |
